# Understanding brain function in vascular cognitive impairment and dementia with EEG and MEG: A systematic review

**DOI:** 10.1016/j.nicl.2022.103040

**Published:** 2022-05-10

**Authors:** Lucía Torres-Simón, Sandra Doval, Alberto Nebreda, Sophia J. Llinas, Elisabeth B. Marsh, Fernando Maestú

**Affiliations:** a Center of Cognitive and Computational Neuroscience; b Department of Experimental Psychology, Cognitive Processes and Speech Therapy, Universidad Complutense de Madrid, Madrid, Spain; c Department of Neurology, the Johns Hopkins School of Medicine, Baltimore, MD USA

**Keywords:** Vascular Cognitive Impairment (VCI), Vascular Dementia (VaD), Neurophysiology, Electroencephalogram, Magnetoencephalogram

## Abstract

Vascular Cognitive Impairment (VCI) is the second most prevalent dementia after Alzheimer’s Disease (AD), and cerebrovascular disease (CBVD) is a major comorbid contributor to the progression of most neurodegenerative diseases. Early differentiation of cognitive impairment is critical given both the high prevalence of CBVD, and that its risk factors are modifiable. The ability for electroencephalogram (EEG) and magnetoencephalogram (MEG) to detect changes in brain functioning for other dementias suggests that they may also be promising biomarkers for early VCI. The present systematic review aims to summarize the literature regarding electrophysiological patterns of mild and major VCI. Despite considerable heterogeneity in clinical definition and electrophysiological methodology, common patterns exist when comparing patients with VCI to healthy controls (HC) and patients with AD, though there is a low specificity when comparing between VCI subgroups. Similar to other dementias, slowed frequency patterns and disrupted inter- and intra-hemispheric connectivity are repeatedly reported for VCI patients, as well as longer latencies and smaller amplitudes in evoked responses. Further study is needed to fully establish MEG and EEG as clinically useful biomarkers, including a clear definition of VCI and standardized methodology, allowing for comparison across groups and consolidation of multicenter efforts.

## Highlights

• Vascular Cognitive Impairment (VCI) is the second most prevalent dementia worldwide.
• Cerebrovascular disease is a major comorbid contributor to neurodegenerative diseases.
• VCI patients show specific spectral, connectivity and evoked responses patterns.
• Literature suggests that EEG-MEG might provide promising biomarkers for early VCI.
• Further neurophysiological research is needed for VCI subtypes differentiation.

## 1 Introduction

The world’s population has increased significantly over the last century as life expectancy has risen from 64.2 years in 1990 to 72.6 years in 2019. Currently, 9% of the population is over the age of 65, and the number of persons aged 80 years or over is projected to triple from 143 million in 2019 to 426 million in 2050 (United Nations, 2019).

Such an increase in life expectancy significantly raises the risk of age-related pathologies, including mild cognitive impairment and dementia. The overall prevalence of dementia is about 2% in people aged 60–69 years, roughly doubling with each 5-year increase in age, reaching up to 66% prevalence in people over 100 years (Cao et al., 2020). There are multiple underlying etiologies, the most common being Alzheimer Disease (AD), which accounts for 60 to 80% of all cases (Alzheimeŕs Association, 2020). Cerebrovascular disease (CBVD) is also a significant cause of cognitive decline in the aging population (Bos et al., 2018, Dey et al., 2016, Kalaria, 2018, Moretti et al., 2011). Second only to AD, it’s estimated that pure Vascular Dementia (VaD) is responsible for 15% of the cases worldwide (Catindig et al., 2012, Kalaria, 2018, O’Brien and Thomas, 2015, Rizzi et al., 2014). In addition, CBVD is also a major comorbid contributor to the progression of other neurodegenerative diseases. Vascular changes are observed in 50%–90% of AD patients (Santos et al., 2017), and in about 50% of other dementia cases worldwide (Wardlaw et al., 2019). Furthermore, the presence of cerebrovascular neuropathology increases the risk of the development of dementia in those with AD neuropathology; but concomitant pathologies skyrocket the risk compared to people with no brain alterations, or even with evidence of exclusively Alzheimer or cerebrovascular-type lesions (Azarpazhooh et al., 2018). Early identification of cognitive decline due to cerebrovascular damage is critically important from the clinical perspective given the opportunity to modify disease progression by controlling risk factors such as hypertension, hyperlipidemia, and diabetes, and treating vascular underlying pathologies (Erkinjuntti et al., 2004, Hachinski et al., 1974, Román and Rogers, 2004).

CBVD involves a spectrum of changes involving the cerebral vasculature including white matter disease, infarction, and hemorrhage. The heterogeneity in pathophysiology and subsequent clinical presentation has made vascular dementia historically difficult to characterize, resulting in a lack of consistency in the terminology used to define the syndrome both for clinical and research purposes. Various international research groups (ADDTC, NINDS-AIREN, AHA/ASA, NINDS-CNS, and VASCOG) have worked to define the concepts, classification, and descriptive terminology surrounding vascular-related cognitive impairment, as consensual classification and clear diagnostic criteria are vital for both research and clinical purposes. The Vascular Impairment of Cognition Classification Consensus Study (VICCCS), published in 2017, is now widely used given its clinical utility. The term VCI includes all forms of mild to severe cognitive impairment associated with and caused by cerebrovascular diseases (O’Brien et al., 2003, Skrobot et al., 2017). The classification ranges from a mild form of vascular cognitive impairment (MildVCI) to major VCI, also called vascular dementia (VaD). Four types of VaD or major forms of VCI were described in VICCCS: subcortical ischemic dementia (SiVaD), post-stroke dementia (PSD), multi-infarct dementia (MID), and mixed dementias (see Table 1). In addition, descriptive terms for either the “mechanism” or “location” of the damage were also defined, including familial/sporadic, strategic infarct, hypoperfusion, hemorrhagic, specific arteriopathies (including genetic, hereditary, and developmental anomalies) and vasculitis.

**Table 1:** Summary of Vascular Cognitive Impairment definition and classification according to VICCCS (Skrobot et al., 2017, Skrobot et al., 2018).

**Mild VCI:** Impairment of at least one cognitive domain with mild to no impairment in instrumental activities of daily living (IADLs)/activities of daily living (ADLs), respectively (independent of the motor/sensory sequelae of the vascular event).		
**Major VCI or VaD:** Clinically significant deficits of sufficient severity in at least one cognitive domain (deficits may be present in multiple domains) and severe disruption to IADLs/ADLs (independent of the motor/sensory sequelae of the vascular event).	**Subcortical Ischemic Dementia (SiVaD)**	Small-vessel disease and white matter lesions are the primary cause of SiVaD. Lacunar infarcts are the most common vascular lesions identified and are located predominantly in subcortical gray and white matter. This diagnosis incorporates the overlapping clinical entities of Binswanger’s disease and the lacunar state.
	**Multi-Infarct dementia (MID)**	MID indicates the presence of multiple large cortical infarcts.
	**Post-Stroke Dementia** **(PSD)**	PSD encompasses dementia that develops within six months of a stroke. There can be multiple cortical-subcortical infarcts or a single strategic lesion. The temporal relationship between cognitive decline and stroke differentiates PSD from other forms of major VCI (VaD).
	**Mixed dementias** **(VCI-another dementia)**	Mixed dementia includes phenotypes representing combinations of vascular and neurodegenerative disease. The most prevalent combination is VCI-AD. This term, describing the pathologies, is now preferred to the previously used but less-specific term “mixed dementia.”
*“Probable” and “possible” VCI	Probable mild VCI or major VCI (VaD) is the appropriate diagnostic category if computed tomography imaging is the only imaging available. Possible mild VCI or major VCI (VaD) is diagnosed when neither MRI nor computed tomography imaging is available.	

Table 1 shows a brief summary of Vascular Cognitive Impairment definition and classification attending to VICCCS. VCI: Vascular Cognitive Impairment; VaD: Vascular Dementia; IADLs: Instrumental Activities of Daily Living; ADLs: Activities of Daily Living; SiVaD: Subcortical Ischemic Dementia; MID: Multi-Infarct dementia; PSD: Post-Stroke Dementia; AD: Alzheimer’s Disease; MRI: Magnetic Resonance Imaging.

The lack of consensus in diagnosis criteria for VCI over the years has impeded sharing and comparison of data on a larger scale (Skrobot et al., 2017). To overcome this challenge, in 2018, VICCCS-2 defined neuropsychological and neuroimaging (i.e., MRI, CT) protocols for diagnosing VCI, gathering and clarifying the previous proposals (Skrobot et al., 2018). However, abnormalities seen on electrophysiological measurements, such as magnetoencephalography (MEG) and electroencephalography (EEG), were not included in these diagnostic criteria due to the lack of consistency. In this systematic review, we evaluate what is currently known regarding the electrophysiological signatures of VCI in an attempt to establish a clear baseline for future research in the field.

### 1.1 Vascular pathophysiology underlying VCI.

To understand the effect of vascular pathology on brain function, we must first consider the Neurovascular Unit (NVU), where the coupling between neural activity and blood flow takes place. The NVU refers to a union of cells of both vascular and neural origin that work together to maintain the homeostatic equilibrium of the brain’s physiological function through autoregulation and hyperemia. The NVU comprises neurons, glial cells (oligodendrocytes, microglia, and astrocytes), vascular cells (endothelial cells, pericytes and smooth muscle cells) and the basal lamina matrix within the vasculature.

Over time, NVU elements undergo multiple aging-related changes, increasing the brain’s sensitivity to ischemia and predisposing it to neurovascular disease (Cai et al., 2017, Yang et al., 2017). Due to aging, there is a progressive failure of the endogenous DNA repair mechanisms in neurons, cytoplasm, and mitochondria-derived proteins, which triggers neuronal oxidative stress and accumulation of toxic proteins such as Amyloid β-peptide (Aβ) (Mattson and Magnus, 2006). Microglia function also declines, dramatically increasing the production of pro-inflammatory molecules and cytokines in response to noxious stimuli (Leovsky et al., 2015, Lourbopoulos et al., 2015). Moreover, damage to myelin and oligodendrocytes exceeds their capacity for repair and renewal, resulting in slower axonal conduction velocity (Peters, 2009, Peters and Sethares, 2004). At the same time, astrocytes show decreased supportive capacity, limiting their regulation of inter-synaptic glutamate concentration, which triggers neuronal excitotoxicity and turns their phenotype into a pro-inflammatory one, inducing blood–brain barrier (BBB) disruption and contributing to brain inflammation. Finally, aging-associated mitochondrial failure affects substance exchange mechanisms and the ability of endothelial cells to regulate cerebral blood flow (Seals et al., 2011), and reduces endothelium-derived vasodilators, which ultimately results in the decrement of the vasodilation capacity (Long et al., 2005, Nicholson et al., 2009, Prisby et al., 2006).

**Fig. 1:**
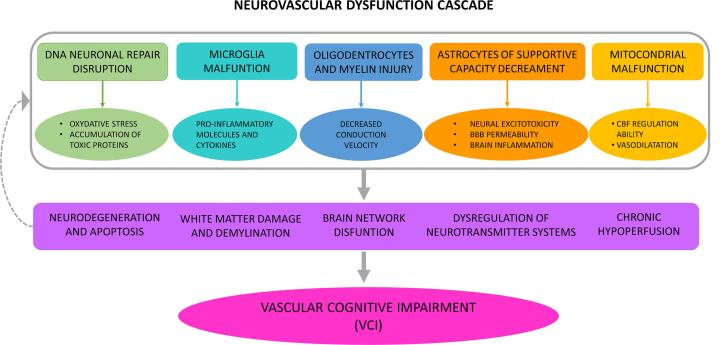
Vascular pathophysiology underlying VCI.

It is proposed that dysregulation or augmentation of these metabolic changes leads to the pathophysiological conditions causative of VCI (see Fig. 1): increased BBB permeability, contributing to neurodegeneration, apoptosis, and functional disruption (Farrall and Wardlaw, 2009, Schreiber et al., 2013, Skoog et al., 1998, Zlokovic, 2008); white matter injuries with axonal damage and even diffuse demyelination (Hase et al., 2018, Ihara et al., 2010, Jang et al., 2017, Kalaria, 2018, Venkat et al., 2017); dysregulation of neurotransmitter systems, such as the cholinergic system (Caruso et al., 2019, Court et al., 2002, Wallin et al., 2003); and alterations of cerebral blood flow and chronic hypoperfusion (Tak et al., 2011, Yang et al., 2002).

### 1.2 Importance of electrophysiology in dementia and mild cognitive impairment

Although structural neuroimaging measures (i.e., MRI or CT) are included within the diagnostic criteria for the VCI, they only partially account for the heterogeneity of behavioral outcomes. Patients with a significant lesion burden can show little to no cognitive impairment, while patients with apparently low vascular lesion load can exhibit significant cognitive deficits. In this sense, metabolic alterations, occurring years before the onset of clinically evident symptoms, may induce some functional changes that cannot be measured with structural measures, but can be well captured using functional neuroimaging techniques as electrophysiology (Dubois et al., 2016, Jiang et al., 2017). Electrophysiological techniques are able to directly capture the electric and magnetic activity generated from post-synaptic activity of apical dendrites in pyramidal neurons (Murakami and Okada, 2006). Specifically, the measurement of the secondary currents in the surface of the head is the basis of EEG, while MEG measures the disturbance of the local magnetic field produced by these currents.

Therefore, electrophysiological techniques result in useful information for assessing brain function and network dynamics, revealing changes inaccessible to standard structural imaging techniques and cognitive assessment. Moreover, in contrast to other functional neuroimaging techniques, they provide information about brain oscillations with millisecond precision and are able to directly capture the neural activation (i.e., pyramidal cells activity) instead of relying on indirect measures, which can be altered by vascular system failures, such as functional Magnetic Resonance Image (fMRI) or Positron Emission Tomography (PET). Moreover, electrophysiological tools allow repeated measurements without any risk for the subjects, as they are non-invasive. Peripheral vascular measures (e.g., blood pressure or heart rate) can also underestimate the effects of CBVD on brain activity during early stages, as they are indirect measurements of systemic vasculature performance.

In part due to the aforementioned advantages, the study of electrophysiological brain signatures (i.e., EEG and MEG) has been well established for early detection and prognosis of other neurodegenerative disorders (López-Sanz et al., 2019) including Alzheimer disease (López-Sanz et al., 2018, Moretti, 2016, Nakamura et al., 2018, Nakamura et al., 2017), Parkinson disease (Stoffers et al., 2008), Lewy body dementia (Matar et al., 2019), and mild cognitive impairment (López-Sanz et al., 2017, Maestú et al., 2015, Pusil et al., 2019). It is reasonable to assume that unique patterns may also exist for VCI and each of its subtypes, and that their identification may also be useful when assessing the possible comorbidity of CBVD with other dementias.

### 1.3 Brain oscillation/rhythms alteration due to pathophysiology in VCI.

EEG and MEG are accurate and non-invasive tools with high temporal resolution, which provide useful information to assess brain function and network dynamics in different aging-related pathologies. Interestingly, the pathophysiology underlying VCI triggers changes in NVU elements closely related to neuronal electrophysiological functioning. Therefore, the biochemical alterations that occur in VCI are capable of modifying cell membrane polarity, action potentials and cell-to-cell communication, thus disturbing the aforementioned neuronal electrophysiological functioning. Those changes in the NVU elements increase the brain’s sensitivity to ischemia and predispose it to chronic hypoperfusion, neurodegeneration and apoptosis, which undoubtedly disturb brain network dynamics as it induces gray and withe matter atrophy. Furthermore, cortical activity depends on a complex balance among different systems of neurotransmitters, and failure within the NVU triggers dysregulation of neurotransmitter function. Lastly, spike timing is vital for proper communication between neurons, and the loss of myelin is known to cause reduction of the speed conduction, disrupting connectivity on both a micro- and macroscopic scale.

The effects of VCI pathophysiology on different key elements related to electrophysiological brain activity, coupled with the ability of EEG and MEG to detect electrophysiological disruptions of the brain, suggest that they could be used as promising biomarkers for early VCI. The development of electrophysiological research in this context could allow not only a better understanding of the progression of the disease, but also the identification of important differences between VCI and AD underlying mechanisms and the discovery of potential treatment targets.

The high prevalence of cerebrovascular disease, along with its modifiable risk factors, support the need for a research effort to study neurophysiological methods that could be useful to detect and differentiate early cognitive impairment (Babiloni et al., 2021). The objectives of this review are to clarify whether there is sufficient evidence for the use of electrophysiology to aid in diagnosis and prognostication of VCI in clinical practice, and to determine what additional steps are needed to one day include these methods as complementary information as part of the diagnostic criteria. This review summarizes the literature regarding neurophysiological patterns, measured with EEG and MEG, for mild and major vascular cognitive impairment. To the best of our knowledge, this is the first study to reproduce a thorough literature review including MEG studies, and also the unique following a systematic review methodology (including PICO search strategy, following the PRISMA guidelines, using a specific methodology for the data synthesis without metanalysis -SWiM, quality assessment - BIOCROSS, and completing the Prospero registration).

## 2 Methods

### 2.1 Literature search

A systematic search of the literature was conducted in September 2020 across PubMed, Cochrane, Web of Science and PsycInfo databases using the PICO search strategy (Miller and Forrest, 2001). Keywords included “vascular dementia“ OR “vascular cognitive impairment” OR “vascular cognitive disorder“ OR “cerebrovascular disease” OR “cerebrovascular disorder“ OR “multi-infarct dementia” OR “subcortical ischemic dementia” OR “post-stroke dementia” OR “mixed dementias” OR “mild vascular cognitive impairment”) AND (“EEG” OR “electrophysiology” OR electroencephalogra* OR “MEG” OR magnetoencephalogra* OR “neural oscillation” OR “brain oscillation” but NOT epilep* [Title/Abstract].

### 2.2 Article’s inclusion and exclusion criteria

Articles meeting the following criteria were included: 1) articles must be written in English and 2) must be peer-reviewed and published in journals indexed in journal citations reports (JCR) since January 2000 (for a review that includes older papers see (Babiloni et al., 2021). Study participants included patients aged 60 years or older who were diagnosed with vascular cognitive impairment (either mild or major VCI). In order not to be too restrictive, we accepted any diagnostic criteria indicative of VCI. Studies also had to report EEG or MEG data and include neuropsychological assessment and/or MRI as diagnostic criteria. Since epilepsy events might affect the establishment of an accurate criterion for VCI diagnosis, articles focused on epilepsy and those focused on treatment evaluation were excluded. Familial pathologies were excluded as not being the scope of this review. Finally, according to the American Psychiatric Association’s Diagnostic and Statistical Manual of Mental Disorders (American Psychiatric Association, 2013) and VICCCS-2 guidelines (Skrobot et al., 2018), the final diagnosis of dementia should be delayed until at least six months after stroke. For that reason, articles reporting post-stroke patients in the acute phase were excluded, as the aim of this review is to characterize diagnostic criteria for VCI and its subtypes.

### 2.3 Screening protocol

The review was registered in PROSPERO CRD42020152953 to avoid duplication and followed a systematic review protocol to ensure the reliability of the process (Moher et al., 2014, Stewart et al., 2012). Articles were imported into COVIDENCE (Veritas Health Innovation), Two reviewers (SD and LT) conducted the review process as recommended in the Preferred Reporting Items for Systematic Reviews and Meta-Analyses (PRISMA) guidelines for systematic reviews (Moher et al., 2010). In the first stage, the article’s abstracts were independently screened according to the established inclusion and exclusion criteria. The full texts of the selected articles were obtained and subsequently reviewed. Disagreements were resolved by expert’s meetings (screening and final selection protocols are depicted in Fig. 3).

### 2.4 Quality assessment

Three reviewers (LT, SD and AN) independently assessed the quality of the selected articles using the Tool for cross-sectional studies using biomarker data (BIOCROSS) (Wirsching et al., 2018). The specific assessment of the items was adapted, as some items could not be easily applied due to the nature of the research field and electrophysiological biomarkers. These specifications did not modify the scale structure or the aim of study for each item. The changes sought to clarify the quality standards according to the study population (i.e., VCI diagnosis criteria) and neurophysiology technical specifications, research protocol or data processing and modeling. (For more details, see “Quality Assessment” in Appendix A). The review was conducted in two rounds. After the first evaluation, the reviewers met to discuss their scoring and addressed potential discrepancies. However, the results were nearly identical across the two rounds. The analysis of both rounds (pre and post) with intra class correlation coefficient (ICC) across the 3 reviewers found significant (p value ≪ 0.05) high ICC scores. Originally, inter-reviewer consistency reached an ICC = 0,811 (95% CI: 0.692–0.895). After discussing the differences and reaching consensus, the ICC raised up to 0,969 (95% CI: 0.946–0.984). No articles were excluded based on the quality assessment (final scores for the 32 articles for each quality item are reported in Appendix Table 1).

### 2.5 Data extraction

The 32 articles were presented in different tables according to the type of analysis performed (visual and spectral analysis, Table 2; connectivity*,* Table 3; and evoked response and entropy, Table 4). In these tables the most relevant information was extracted for each of the articles, following the same structure:

***Authors & Publication date:*** First author and year of publication.

***VCI Subtype:*** We listed the VCI subtype corresponding to population diagnosis under the VICCCS-2 criteria. Independent of the nomenclature used in each paper for the subtype of dementia, we categorized each article in the VICCCS subtypes according to MRI/CT data or detailed clinical diagnosis when reported. Patients were classified as: subcortical ischemic vascular dementia (SiVaD) when they displayed white matter hyperintensities (WMH) on neuroimaging; post-stroke dementia (PSD) when lacunar subcortical or/and large-vessel strokes with subsequent cognitive impairment was described; multi-infarct dementia (MID) when several cortical infarcts were evidenced; and mixed dementia, when signatures of two different pathophysiology were defined (i.e., atrophy and WMH). When classification was not possible, we reported them as non-determined “N/D VCI”.

***Sample characteristics:*** Including diagnostic criteria for each group (according to each paper nomenclature), number of subjects, sex, and age.

***Diagnosis***: MMSE and MRI/CT (we describe objective measures for VCI diagnosis when the authors report them in the original article).

***Methods***: Neuroimaging technique, experimental condition, and type of signal analysis.

***Main Results:*** Were briefly described for each article*.*

### 2.6 Data synthesis

**Fig. 2:**
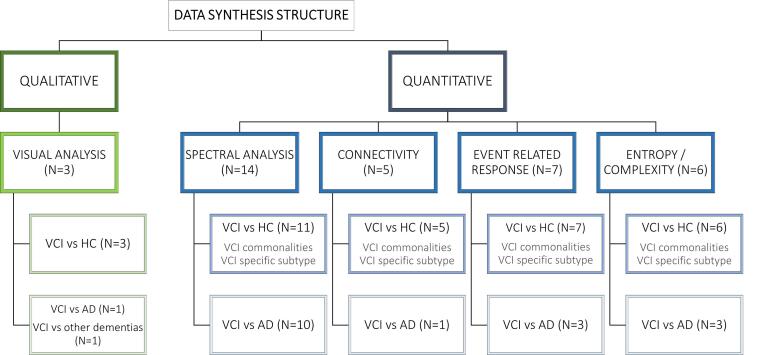
Data synthesis structure.

Often in systematic reviews, *meta*-analysis is not possible or appropriate due to the incomplete reporting of effects or because of the characteristics of the selected studies (design, population, experimental condition, or data analysis). In these reviews, in order to be clear and rigorous, alternative synthesis methods may be adopted. Due to the diversity in subtypes of dementia, severity, recording conditions, and research and analysis methods, there was no simple way to aggregate the results. We therefore employed the synthesis without *meta*-analysis (SWiM) guidelines (Campbell et al., 2020). In this context, results are initially divided according to the type of analysis: qualitative analysis, based on visual inspection and interpretation of the recording by a panel of experts, and quantitative analysis, based on objective numerical analysis of the data. Quantitative analysis can be further subdivided into: spectral analysis, which quantifies the amount of oscillatory activity of different frequency in the signal; functional connectivity, which assesses statistical associations between different spatial areas; event-related potentials (ERPs), which measure the averaged waveform following a specific event; and entropy and complexity, which measure the non-linear properties of the signal related to the amount of uncertainty, randomness or level of complexity of the signal (see Fig. 2).

For each type of analysis, we separated the results according to the groups that were compared: 1) studies comparing VCI vs. healthy controls (HC); and 2) studies comparing VCI and AD. In addition, results were listed according to population diagnosis under the VICCCS-2 criteria. Regardless of the nomenclature and diagnosis criteria used in each paper for the subtype of dementia, we categorized each article in the VICCCS subtypes according to MRI/CT data or detailed clinical diagnosis when reported, resulting in five groups: 1) “*VCI commonalities*'' between subtypes of VCI. Under the heading “*VCI commonalities*”, we described the results that were indifferent to the VCI subtypes framework. This may occur because the results were common across two or more subtypes or because the subtypes were not clearly defined. In subtype-specific categories, we reported results that were described only for each corresponding subtype. 2) “*SiVaD*”; 3) “*PSD*”; 4) “*mixed dementia*” (VCI-AD); and 5) “*MID*”. MID classification was later dropped when no appropriate articles were found (see Fig. 2). It is important to note that none of the papers included in the present systematic review conducted comparisons between different subtypes of VCI. Therefore, although we have divided the results according to different subtypes of VCI, it would not be appropriate to assume that patterns found are unique or differential for each subpopulation without further research directly comparing different subtypes.

Finally, the severity of the disease was assessed in several ways across studies, mainly using a cognitive measure such as the MMSE or other neuropsychological tests, but sometimes by directly assessing the severity of the anatomical damage shown by structural neuroimaging techniques (MRI or CT).

## 3 Results

**Fig. 3:**
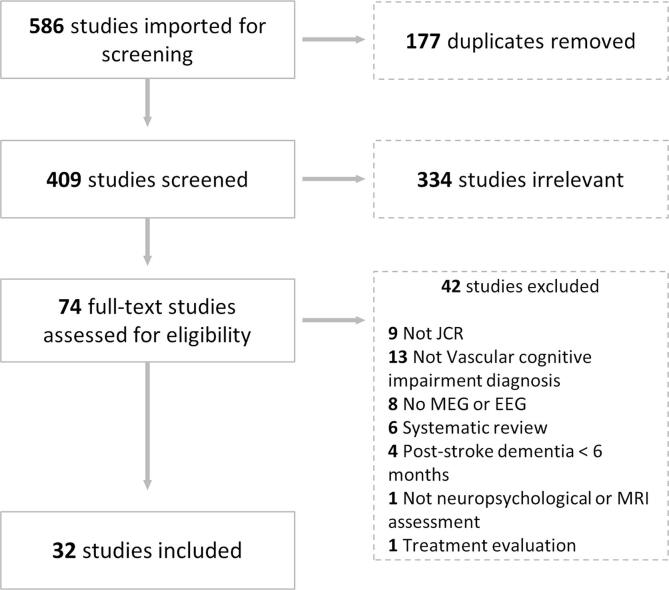
Flowchart of included and excluded articles through the screening process following the PRISMA presentation guidelines.

A total of 586 articles were imported for screening after conducting a literature search in the specified datasets. After removing those duplicated and those identified as irrelevant for the aim of this systematic review in the abstract screening, a total of 74 studies were assessed for eligibility, and their full text were reviewed. Based on our exclusion criteria, 42 studies were excluded: nine of them were not JCR; thirteen did not include a vascular cognitive impairment diagnosis; eight did not perform MEG or EEG; six were systematic reviews four included a vascular dementia diagnosis less than six months post-stroke; one did not use neuropsychological or MRI assessment; and one focused only on treatment evaluation. Thirty-two studies were included for further analysis in this systematic review (see Fig. 3).

### 3.1 Visual analysis

Only 3 articles were included in this category; all of them based on EEG signal analysis. Only one exclusively reported visual analyses comparing patients with VCI to HC and other dementias (Liedrop et al., 2009). The other two also conducted spectral analysis, Gawel et al. (2007) comparing VCI to AD and HC; Gawel et al. (2009) only to HC. Specific Information for these articles is detailed in Table 2.

#### 3.1.1 VCI vs. HC

On visual analysis, we could conclude that more abnormalities, both focal slow or sharp wave activity and diffuse slow-wave activity, were reported for VCI patients than for healthy age-matched controls (Gawel et al., 2009, Gawel et al., 2007, Liedrop et al., 2009). No significant association between visually assessed abnormalities and the severity of dementia evaluated by MMSE was found (Gawel et al., 2009, Gawel et al., 2007).

#### 3.1.2 VCI vs. other types of dementia

Based on the single study included in this systematic review performing exclusively visual analysis, evaluations of EEG signals during resting state were divided into four categories (“normal EEG”, “focal abnormalities”, “diffuse abnormalities”, and “both focal and diffuse abnormalities”). This classification was performed considering the criteria that the authors reported: “*Focal abnormality was defined as (transients of) slow or sharp wave activity in 1 or more EEG leads, including epileptiform abnormalities, but excluding benign temporal theta of the elderly. A dominant frequency of rhythmic background activity below 8 Hz, diffuse slow-wave activity, and diminished reactivity of the rhythmic background activity to the opening of the eyes were each considered a criterion for a diffuse disturbance”* (Liedrop et al., 2009).

According to this classification, researchers found that a “normal EEG” was more common in HC and subjects with psychiatric disorders (Psych) and less common in patients with VCI, AD and dementia with Lewy bodies (DLB). “Focal abnormalities” alone were more common in mild cognitive impairment (MCI) and less common in DLB. “Diffuse abnormalities” alone were more common in AD and less common in HC. “Both focal and diffuse abnormalities” were more common in VCI, AD and DLB, and less common in HC, Psych and MCI (Liedrop et al., 2009). When comparing exclusively AD and VCI with similar cognitive impairment, fewer slow waves were found in the VCI group, both in mild and moderate dementia (Gawel et al., 2009, Gawel et al., 2007).

### 3.2 Spectral analysis (Table 2)

Fourteen articles were included in this section; 11 describing results of VCI compared to HC, 10 comparing VCI to AD, and most reporting comparisons between all three groups. Several methodologies were used to analyze the signal spectrum: evaluating the relative power of the signal in each frequency band, the ratios between the power found in different bands, the α peak’s amplitude, frequency and dispersion, the symmetry of the power distribution across the brain, and the displacement of the general frequency of the spectrum. For all articles, the physiological signal was recorded using EEG during the resting-state, with the exception of three: Tsuno et al. (2004), Xu et al. (2011), and Al-Qazzaz et al. (2017b), that conducted the recordings during the transition from alertness to sleep, a visual task, and an auditory task respectively.

**Table 2:** Summary of studies assessing visual and spectral analysis.

Table 2 shows a summary of those studies that assess visual and spectral analysis. Including authors reference, VCI subtype according to VCCCI, sample characteristics, subjects’ diagnosis, methodology used, and a brief summary of their main findings. Color blue represents power decrements whereas red color represents increments. VCCCI: Vascular Impairment of Cognition Classification Consensus Study; EEG: Electroencephalogram; MEG: Magnetoencephalography; VCI: Vascular Cognitive Impairment; N/D VCI: Non-Determined VCI subtype; N/R: Non-Reported; VaD: Vascular Dementia; AD: Alzheimer’s Disease; DBL: Dementia With Lewy Bodies; MCI: Mild Cognitive Impairment; Mild AD: Mild Alzheimer Disease; SIVD: Sub-Cortical Ischemic Vascular Disease; FTLD: Frontotemporal Lobar Degeneration; Psychiat: Psychiatric Disorder; SC: Subjective Complaints; EC: Eyes-Closed; EO: Eyes-Open; MRI: Magnetic Resonance Imaging; CT: Computed Tomography; SiVaD: Subcortical Ischemic Dementia; SVD: Subcortical Vascular Dementia; SVD I: Mild Dementia; SVD II: Moderate Dementia; AD I: Mild Dementia; AD II: Moderate Dementia; AD III: Marked Dementia; CV: Cerebrovascular; mildCV: Mild Vascular Damage; moderateCV: Moderate Vascular Damage; severeCV: Severe Vascular Damage; vCIND: Vascular Cognitive Impairment, No Dementia; amdMCI: Amnesic Multidomain Mild Cognitive Impairment; TIA: Transient Ischemic Attack; HC: Healthy Controls; WMH: White Matter Hyperintensities; ARWMC: Age-Related White Matter Changes; NINDS-AIREN: National Institute Of Neurological Disorders And Stroke And Association Internationale Pour La Recherché Et l’Enseignement En Neurosciences; NINCDS-ADRDA: National Institute Of Neurologic, Communicative Disorders And Stroke - Alzheimer's Disease And Related Disorders Association; ICD-10: International Classification Disease; DSM-III-R: Diagnostic And Statistical Manual Of Mental Disorders (1987); DSM-IV: Diagnostic and Statistical Manual of Mental Disorders-4th Edition; NIHSS: National Institutes of Health Stroke Scale; RAVLT: Rey Auditory Verbal Learning Test; MMSE: Mini-Mental State Examination; CDR: Clinical Dementia Rating Scale; CAMDEX: Cambridge Mental Disorders of the Elderly Examination; DTABR: (delta 1 theta)/(alpha 1 beta) ratio; α/δ: alpha/delta power ratio; α/θ: alpha/theta power ratio; TF: Transition Frequency; IAF: Individual Alpha Frequency; pdBSI: Pairwise Derived Brain Symmetry.

#### 3.2.1 VCI vs. HC

##### 3.2.1.1 VCI commonalities

On spectral analysis, when comparing VCI patients to HC, a generalized pattern of diffuse slowing was most often described. Increased slow rhythms in delta (δ) and theta (θ) bands were repeatedly found in VCI patients (Al-Qazzaz et al., 2017b, Al-Qazzaz et al., 2017a, Babiloni et al., 2004a, Moretti et al., 2004, Neto et al., 2015, Schreiter Gasser et al., 2008, van Straaten et al., 2012, Wu et al., 2014). Greater severity of the disease (assessed with MRI and MMSE) was strongly associated with higher power in both δ and θ bands (Al-Qazzaz et al., 2017b, Al-Qazzaz et al., 2017a, Moretti et al., 2007).

Greater variability was seen in the alpha (α) band, likely due to differences in the band frequencies definition. While some authors took the entire α band as a whole, others divided it into either two or three sub-bands (α1-low, α2-medium, α3-high). The precise definition for the alpha band and sub-bands for each study can be found in Table 2. Neto and collaborators (2015) found a greater α peak amplitude in patients with VCI than in controls, although the peak frequency was found to be greatly slowed (mean was around 8 Hz for VCI). Alternatively, van Strateen et al. (2012) found lower α power, especially in posterior regions, for VCI patients compared to HC. Babiloni et al. reported in 2004 lower posterior low-α power in VCI patients, which has also been negatively associated with cerebrovascular damage severity and cognitive performance in many articles (Moretti et al., 2007, Sheorajpanday et al., 2013, Wu et al., 2014). Interestingly, in one of the studies, VCI subjects with mild disease severity exhibited higher low-α power than controls (Al-Qazzaz et al., 2017b). In this last study, it is important to note that the recordings consisted of an auditory task instead of a resting-state activity, and that the sample size of the VaD sample was only five patients. Therefore, results must be considered appropriately. Regarding high-α results, lower high-α was found for VCI patients compared with HC, which was again associated with increased severity of dementia (Al-Qazzaz et al., 2017b, Moretti et al., 2004). Finally, studies evaluating peak frequency reported a slower α peak frequency in VCI patients compared to HC (Moretti et al., 2004, Neto et al., 2015), and greater occipitotemporal dispersion of the α peak (Neto et al., 2015).

When evaluating the beta (β) band, studies uniformly showed decreased power in VCI patients compared with HC, once again associated with greater evidence of cognitive symptomatology (Al-Qazzaz et al., 2017b, van Straaten et al., 2012).

##### 3.2.1.2 Subcortical Ischemic vascular dementia (SiVaD)

Focusing on the articles evaluating patients with SiVaD, results were similar to patients with a general non-determined subtype of VCI (increased slow waves and decreased alpha power). Power ratio abnormalities were reported by three different articles. They found lower α/θ power ratio (Gawel et al., 2009, Gawel et al., 2007, Moretti et al., 2007), lower α/δ and α/(θ + δ) (Gawel et al., 2009, Gawel et al., 2007) and lower low-α/high-α (Moretti et al., 2007) ratios, all of them related to the severity of the disease. Additionally, a higher left–right power asymmetry was found to be related to greater subcortical ischemic damage (Sheorajpanday et al., 2014), as well as an abnormal (widespread) source distribution in θ band (Babiloni et al., 2004a). Finally, a general decrement in the mean frequency (Gawel et al., 2009, Gawel et al., 2007) and a slower θ to α transition were explicitly found in SiVaD patients (Moretti et al., 2007), again associated with greater disease severity (Gawel et al., 2009, Gawel et al., 2007, Moretti et al., 2007). In other vein, during sleep onset, the changes in the intracerebral EEG main generator over time were studied, finding that, on the high-α band, the fluctuations of the generator along the superior-inferior axis of the brain were larger in VCI than in HC (Tsuno et al., 2004).

##### 3.2.1.3 Post-stroke dementia (PSD)

Only two studies evaluated the spectral analysis in patients with PSD. Both included task conditions; one auditory (Al-Qazzaz et al., 2017b), and one visual (Xu et al., 2011). In this context, results aggregation was not convenient as experimental conditions were entirely different. Furthermore, these studies were unique, as patients were recorded while performing the task instead of the more common task-free paradigm. Therefore, results could be task-related, rather than specific to the VCI subtype.

For the visual task study, power was defined as the square of the amplitude samples; however, the data were analyzed using the event-related synchronization/desynchronization (ERS/ERD) method. PSD patients showed decreased event-related synchronization (ERS) in the δ frequency band in the frontal, central and parietal areas. Moreover, they detected a similar pattern for elderly healthy controls compared to young HC (Xu et al., 2011).

For the auditory task study, along with changes in the slower bands, an augmentation in gamma (γ) power was described for PSD patients, showing a direct relationship with increased disease severity (Al-Qazzaz et al., 2017b). The same study assessed power ratios of different frequency bands, finding that PSD patients presented with higher low-α/high-α, higher θ/γ, and lower high-α/β1 than controls, with this pattern associated with the severity of the disease. There appears to be a different behavior in the low-α/high-α power ratio between SiVaD (Moretti et al., 2007) and PSD (Al-Qazzaz et al. 2017b), which could be used in the future to distinguish between them. However, this requires further investigation directly comparing both groups of patients and also further replication studies to ensure this differentiation.

##### 3.2.1.4 Mixed dementia

Only one study included a sample of mixed VCI-AD dementia patients. They found less topographical differences between anterior and posterior distributions for high-α, β1 and β2 power, similar to that seen in pure AD patients (Schreiter Gasser et al., 2008).

#### 3.2.2 VCI vs. AD

While slow waves were commonly increased in AD compared to HC, VCI patients displayed even higher power than AD patients with similar severity, in both δ and θ bands (Babiloni et al., 2004a, Moretti et al., 2007, Neto et al., 2015, Schreiter Gasser et al., 2008, Sheorajpanday et al., 2013, Wang et al., 2014). However, one of the studies found lower δ and θ power in VCI than in AD with similar cognitive impairment measured with MMSE (Wu et al., 2014). Regarding the α band, the results depicted a more complex picture. When comparing VCI with AD patients, there was a greater α peak amplitude for VCI patients, although the mean peak was found around 8 Hz in both groups (Neto et al., 2015). When assessing power, both lower (Sheorajpanday et al., 2013) and higher (Babiloni et al., 2004a) low-α power have been reported. Finally, also higher high-α power in the VCI group when compared to AD patients has been described (Moretti et al., 2007, Moretti et al., 2004, Schreiter Gasser et al., 2008).

When evaluating power ratios in different frequency bands, VCI patients (specifically SiVaD) displayed higher α/δ, α/θ and α/(θ + δ) than AD patients (Gawel et al., 2009). A seemingly opposite pattern was also found, with a lower (α + β)/(δ + θ) power in SiVaD, which the authors justify by the inclusion of the β band when calculating the ratio (Sheorajpanday et al., 2014).

In comparison to AD, VCI patients displayed a higher power asymmetry (pdBSI) (Sheorajpanday et al., 2014), as well as an abnormal source distribution in θ band (Babiloni et al., 2004a). During sleep onset, within the high-α band, the fluctuations of the main signal generator along the superior-inferior axis of the brain were larger in VCI than in AD patients. In contrast, no significant differences in this or other axis fluctuations were reported for AD compared to HC (Tsuno et al., 2004).

Finally, a slower α peak frequency was found for VCI -than for AD in frontal and frontotemporal areas (Moretti et al., 2004, Neto et al., 2015), as well as lower dispersion of the α peak in temporal areas (Neto et al., 2015). Moreover, lower θ to α transition frequency was found in VCI compared to AD patients (Moretti et al., 2007). Furthermore, VCI and AD patients presented a greatly decreased average frequency in occipital areas, while AD patients also showed a lower average frequency in temporal lobes, perhaps hinting at different patterns of structural degeneration (Gawel et al., 2009).

### 3.3 Connectivity analysis (Table 3)

Five articles were included in this section; 2 recorded during resting-state (Babiloni et al., 2004b, van Straaten et al., 2015) and 3 during visual tasks (Wang et al., 2016, Wang et al., 2014, Xu et al., 2015). All of the studies performing connectivity analyses were measured using EEG, and calculated directly between sensors, without performing source reconstruction. Connectivity analyses reported in these studies were heterogeneous in methodology, evaluating both functional connectivity (non-directional, statistical dependencies among neurophysiological signals), with techniques such as Synchronization Likelihood (SL) or Phase Lag Index (PLI); as well as effective connectivity (directional influence that a node exerts over another under a network model of causal dynamics), using directed Phase Lag Index (dPLI), Directed Transfer Function (DTF), or short Directed Transfer Function (sDTF). Additionally, some manuscripts used network analysis methods: clustering coefficient (C_p_), characteristic path length (L_p_), in-degree (number of incoming connections) and out-degree (number of outcoming connections), considering hubs those nodes with a higher (one SD in this specific paper) in-degree or out-degree than the average. While acknowledging that these metrics do not measure the same characteristics, we have tried to integrate the information in the most meaningful way; however, it is important to consider that this could easily lead to potential contradictions.

**Table 3:** Summary of studies assessing connectivity analysis.

Table 3 includes a summary of those studies that assess connectivity analysis. Including authors reference, VCI subtype according to VCCCI, sample characteristics, subjects’ diagnosis, methodology used, and a brief summary of their main findings. Color blue represents decrements report whereas red color represents increments, regarding connectivity analysis parameters. VCCCI: Vascular Impairment of Cognition Classification Consensus Study; EEG: Electroencephalogram; MEG: Magnetoencephalography; SiVaD: Subcortical Ischemic Dementia; VaD: Vascular Dementia; PSD: Post-stroke dementia; AD: Alzheimer’s Disease; Mild AD: Mild Alzheimer Disease; NINDS-AIREN: National Institute Of Neurological Disorders And Stroke And Association Internationale Pour La Recherché Et l’Enseignement En Neurosciences; NINCDS-ADRDA: National Institute Of Neurologic, Communicative Disorders And Stroke - Alzheimer's Disease And Related Disorders Association; HC: Healthy Controls; WMH: White Matter Hyperintensities; DSM-IV: Diagnostic and Statistical Manual of Mental Disorders-4th Edition; SL: Synchronization Likelihood; PLI/dPL: Phase Lag Index/ directed phase lag index; DTF: Directed Transfer Function; sDFT: Short Directed Transfer Function.

#### 3.3.1 VCI vs. HC

##### 3.3.1.1 VCI commonalities

The most common and prominent effect described was decreased connectivity in slow bands between parietal and frontal areas, found in δ (Babiloni et al., 2004b, van Straaten et al., 2015, Wang et al., 2016, Xu et al., 2015), θ (van Straaten et al., 2015, Wang et al., 2016, Wang et al., 2014, Xu et al., 2015), and low-α bands (Babiloni et al., 2004b, van Straaten et al., 2015, Wang et al., 2016, 2014). The most affected area appeared to be the parietal lobe, which showed loss of an out-degree hub (number of outcoming connections) with respect to HC (Wang et al., 2016, 2014). Interhemispheric connectivity abnormalities were also commonly observed in slow bands (Babiloni et al., 2004b, Xu et al., 2015).

##### 3.3.1.2 Subcortical Ischemic vascular dementia (SiVaD)

In a resting state study assessing FC, an all-band decrement of interhemispheric connectivity (using SL) was found in patients with subcortical ischemic damage compared to HC (Babiloni et al., 2004b). Additionally, a consistent front-to-back pattern of phase relations (using dPLI) in all bands except δ have been described for HC, but this pattern was not present in SiVaD patients and was even reversed in the β band (van Straaten et al., 2015). No significant relationships were found between cognitive performance and connectivity patterns (using either PLI or dPLI) (van Straaten et al., 2015).

##### 3.3.1.3 Post-stroke dementia (PSD)

The three studies evaluating PSD patients were based on visual oddball paradigm tasks. Connectivity was most dramatically affected within the parietal regions for patients with PSD compared to HC. Wang et al. in 2014 studied the dynamical change of connectivity (using sDTF) at different time points after the presentation of a visual stimulus and found that the primary links affected were those originating in the medial parietal lobe, for δ, θ and low-α bands, and, to a lesser extent, from frontal areas in the θ band. Wang et al. in 2016, using the same visual oddball paradigm, found that while controls had three main out-degree hubs in δ (medial frontal, medial central and medial parietal), PSD patients lacked the one in the medial parietal lobe. In addition, they found abnormalities in the medial central region, with a lower δ, θ & β out-degree, and higher δ & β in-degree. PSD patients also showed decreased C_p_ in δ, θ and low-α bands, suggesting a decreased tendency to form densely connected clusters (Wang et al., 2016, 2014). Alterations in the δ and θ bands connectivity (using DTF) were also found, especially in δ, pre- and post-stimulus in PSD patients compared to HC (Xu et al., 2015). Pre-stimulus, most abnormalities seemed to be interhemispheric, from left to right, but, post-stimulus, an important reduction of connectivity from parietal to other regions appeared in δ, especially to frontal areas (Xu et al., 2015).

#### 3.3.2 VCI vs. AD

Only one paper assessed differences between patients with VCI (specifically SiVaD) and AD (Babiloni et al., 2004b). While both VCI and AD patients showed connectivity decrements compared to HC, those with VCI depicted lower low-α interhemispheric connectivity (using SL) compared to AD patients. Additionally, while those with AD exhibited less intense reduction at frontal than seen at parietal electrodes, VCI patients showed a homogeneous decrease over the whole scalp).

### 3.4 Event-related response (Table 4)

Seven articles were included in this section. Three studies were based on visual tasks recorded with EEG (Beuzeron-Mangina and Mangina, 2009, Rosengarten et al., 2007, Xu et al., 2012), 3 on auditory tasks, also with EEG (Jiang et al., 2017, Van Harten et al., 2006, Yamaguchi et al., 2000), and 1 on auditory and somatosensory tasks recorded with MEG (Sun et al., 2013). The literature on ERP was highly heterogeneous with respect to experimental conditions, events considered in each study, and types of analyses, so aggregating the results was a challenging task, consequently results generalization should be done with special caution.

**Table 4:** Summary of studies assessing evoked response and entropy/complexity analysis.

Table 4. includes a summary of those studies that assess evoked response and entropy/complexity analysis. Including authors reference, VCI subtype according to VCCCI, sample characteristics, subjects’ diagnosis, methodology used, and a brief summary of their main findings. Color blue represents decrements report whereas red color represents increments, regarding entropy/complexity parameters. VCCCI: Vascular Impairment of Cognition Classification Consensus Study; EEG: Electroencephalogram; MEG: Magnetoencephalography; VCI: Vascular Cognitive Impairment; SiVaD: Subcortical Ischemic Dementia; SIVD: Subcortical Ischemic Dementia; VaD: Vascular Dementia; VD: Vascular Dementia; P-VD: Prodromal Stages of VaD; VCI-ND: Vascular Cognitive Impairment with No Dementia; MVD: Mild Vascular Dementia; PSD: Post-stroke dementia; MCI: Mild Cognitive Impairment; AD: Alzheimer’s Disease; VEAD: Very Early Alzheimer's Disease; P-AD: Prodromal Stages Of AD; HC: Healthy Controls; WMH: White Matter Hyperintensities; MMN: Mismatch Negativity; ERS: Event-Related Synchronization; NINDS-AIREN: National Institute Of Neurological Disorders And Stroke And Association Internationale pour la Recherché Et l’Enseignement en Neurosciences; NINCDS-ADRDA: National Institute Of Neurologic, Communicative Disorders And Stroke - Alzheimer's Disease And Related Disorders Association; DSM-IV: Diagnostic and Statistical Manual of Mental Disorders-4th Edition; NIHSS: National Institutes of Health Stroke Scale; MMSE: Mini-Mental State Examination; HDS: Dementia Scale; MRI: Magnetic Resonance Imaging; CT: Computed Tomography; D2: Correlation Dimension; L1: Lyapunov exponents; PerEn: Permutation Entropy; FD: Fractal; SVM: Support Vector Machine; kNN: k-nearest neighbors.

#### 3.4.1 VCI vs. HC

##### 3.4.1.1 VCI commonalities

*Regardless* of the nature of the task (visual or auditory), VCI patients showed common patterns with a smaller P3 amplitude and a longer latency than HC (Van Harten et al., 2006, Xu et al., 2012, Yamaguchi et al., 2000).

##### 3.4.1.2 Subcortical Ischemic vascular dementia (SiVaD)

During auditory tasks, SiVaD patients depicted a smaller N1 amplitude than HC participants for standard tones, but similar latency, while showing a smaller P3 amplitude and longer latency following target and novel sounds (Yamaguchi et al., 2000). A smaller and earlier frontocentral mismatch negativity (MMN) amplitude was also reported for SiVaD patients (Jiang et al., 2017). Additionally, SiVaD patients, compared to HC, were found to have a longer N2 latency, as well as a smaller peak-to-peak amplitude when comparing the N2 complex to the P3 wave. Longer N2 latency was associated with higher disease severity, while the P3 latency was not (Van Harten et al., 2006). In a visual flickering task, a smaller N75-N100 amplitude was found in SiVaD patients (Rosengarten et al., 2007). SiVaD patients also presented no significant differences in P450 amplitudes compared to HC when performing a visual memory task. However, SiVaD patients displayed longer latencies over anterior regions and shorter over posterior, a reversed pattern from the one found in HC (Beuzeron-Mangina and Mangina, 2009).

On MEG, longer latencies, and greater equivalent current dipole (ECD) amplitudes were found for M20 in the somatosensory task, and a longer M100 latency in the auditory task, in their respective primary sensory areas, compared to HC. These characteristics were also associated with greater disease severity (Sun et al., 2013).

##### 3.4.1.3 Post-stroke dementia (PSD)

As only one paper included post-stroke patients in its sample, results could not be aggregated with previous research. The article had a small sample size, so caution should be used when generalizing to the entire VCI population; however, smaller P3 amplitudes and longer latencies were also reported. (Xu et al., 2012).

#### 3.4.2 VCI vs. AD

During auditory tasks, AD and VCI patients exhibited the same pattern of differences compared to HC in P3 amplitude and latency after target tones, but not after novel sounds. No significant differences between AD and VCI were found in N1 after standard tones, nor in P3 amplitude or latency following target tones; however, the P3 amplitude of VCI patients following novel sounds was smaller than that of AD, and the latency longer (Yamaguchi et al., 2000). No significant differences between AD and VCI were found in MMN mean amplitude or latency; both showing the same pattern of differences with HC (Jiang et al., 2017). During a visual flickering task, VCI patients presented lower N75-N100 amplitude than both HC and AD patients (Rosengarten et al., 2007), and in a study using a visual memory workload task, VCI patients depicted lower P450 amplitudes than AD patients (Beuzeron-Mangina and Mangina, 2009).

### 3.5 Entropy and complexity (Table 4)

Six studies evaluated entropy and complexity. All of them used EEG recordings. Two were recorded during visual tasks (Lou et al., 2011, Xu et al., 2012), 1 during an auditory task (Al-Qazzaz et al., 2017a), 2 during resting-state alone (Jeong et al., 2001, Kim et al., 2001), and 1 during resting-state and photic stimulation (Lin et al., 2015). Similar to connectivity analysis, several possible methods can be used to calculate entropy and complexity. Studies in this review used: Approximate Entropy (ApEn), number of forbidden words (NumFW), Sample Entropy (SampEn), correlation dimension (D2), Lyapunov exponents (L1 to Ln), Permutation Entropy (PerEn), Fractal Dimension (FD), and multichannel linear descriptors (Ω, Ф and Σ). Although all of the methods measure non-linear properties related to the complexity of the signal, each of them uses different mathematical approximations to address the topic. The differences between methods are not easily translatable into biological terms, beyond being able to relate them with a greater or lesser complexity of some of the properties of the brain activity. Greater or lesser complexity doesn’t have a unique or easy meaning in a clinical sense, with abnormalities in either direction being a possible marker for disease. For this reason, it is difficult to cluster results in a meaningful way without making assumptions and simplifications. Therefore, we present the results independently so the reader can arrive at their own conclusion.

#### 3.5.1 VCI vs. HC

##### 3.5.1.1 VCI commonalities

Though no common results were found across subtypes, in general VCI patients showed higher D2 and L1 during resting state (Jeong et al., 2001) and less regular symbolic dynamics during both resting state and photic stimulation, represented by lower NumFW and higher SampEn (Lin et al., 2015). This indicates higher entropy and complexity in the dynamic processes underlying the signal. However, in other resting state studies, VCI patients exhibited a lower Wackermann’s Ω complexity, which indicates that results could be explained with a lower number of independent components. The most dominant components, which accounted for more variability of the total signal in VCI than in HC, also varied in their topological distribution, being more significant in postero-temporal areas and less in central and anterior areas (Kim et al., 2001).

##### 3.5.1.2 Post-stroke dementia (PSD)

During an auditory task, PSD patients displayed a lower PerEn and FD than HC in frontal, temporal, and central regions, which were lower as disease severity increased. Both indexes are associated with more regular and less complex activity (Al-Qazzaz et al., 2017a). However, in a visual oddball task, PSD patients showed an increased ApEn for PSD during the response period, associated with more complex activity and possibly less synchronous waveforms in this phase (Xu et al., 2012).

With regards to complexity measures, PSD patients showed, during a visual oddball task, higher Ω and Ф, and lower Σ, in δ band, as well as higher Ω in θ band. Higher Ω and Ф in δ band were associated with increased disease severity, while Σ was not. A higher Ω is usually interpreted as a lower synchronization, while the interpretation of Σ and Ф is less clearly established (Lou et al., 2011).

#### 3.5.2 VCI vs. AD

While VCI patients showed higher D2 and L1 than HC during resting state, indicating higher entropy, AD patients had lower D2 and L1 than both HC and VCI patients in most regions, indicating lower entropy (Jeong et al., 2001). VCI patients also presented less regular symbolic dynamics, associated with higher entropy and complexity, represented by lower NumFW and higher SampEn, than AD patients. This was the case both during resting state and photic stimulation, with the greater differences being found during the stimulation (Lin et al., 2015).

During resting state, no significant differences in general Wackermann’s Ω complexity were found between VCI and severe AD patients. However, VCI patients presented lower Wackermann’s Ω complexity when compared with AD patients with a similar degree of deterioration, assessed using MMSE. The pattern of differences in the distribution of the most dominant components appeared similar when comparing VCI or severe AD to HC, while the differences were milder when comparing moderate AD to HC (Kim et al., 2001).

## 4 Discussion

The heterogeneity within the existing literature with respect to both the study population and methods of analysis makes the generalization of conclusions difficult. In most cases it is not possible to differentiate between VCI subtypes, or to assess the degree of comorbidity in mixed dementia patients. Most of the papers included in this systematic review do not report objective MRI measures to estimate structural vascular damage, or neuropsychological batteries beyond MMSE scores. This illustrates a significant knowledge gap in VCI literature, likely secondary to changes in terminology and the inclusion criteria over time. This heterogeneity, along with relatively small sample sizes, may explain the seemingly contradictory results. The lack of a clear definition of VCI and its subtypes precludes accurately assessing potential differences in electrophysiological signatures between groups. Nonetheless, significant progress has been made, and we are hopeful that similar to the framework developed by international consortiums for AD (Albert et al., 2011, Dubois et al., 2016, McKhann et al., 2011, Sperling et al., 2011), VICCCS will allow for significant future progress and collaboration.

Along with the need for consensus definitions, standard methodologies are also required to advance the field further. This literature review reveals little consistency or replication of results. The different approaches in data analysis hinders the reliability, repeatability, and reproducibility of the results. In addition to the standardization of signal analysis methods, we recommend the incorporation of modern analyses, which could overcome many of the difficulties encountered when using classical methods. In this regard, the novel spectral analysis method proposed by Donoghue et al., (2020), which consists in the parameterization of the neural power spectra into periodic and aperiodic components, deserves special mention. This method has already provided promising results, showing that aperiodic activity changes are strongly associated with aging (Brady and Bardouille, 2022, Thuwal et al., 2021), and with Alzheimer's dementia (Wiesman et al., 2022). Moreover, most of the studies incorporated in this review report data from resting state conditions. While this can be useful for clinical practice (e.g., to identify the patient’s disease), the study of brain function during specific cognitive tasks could also provide meaningful knowledge and warrants further investigation. We encourage investigation of electrophysiological function during resting state to obtain reliable and replicable data that could be used as biomarkers (Colclough et al., 2016, Garces et al., 2016)as well as during cognitive tasks typically impaired in VCI patients, including executive function and processing speed in order to evaluate the evolution of the disease.

Despite the limitations, by analyzing the outcomes of the papers selected for this systematic review, specific patterns did emerge for patients with VCI compared to HC and those with AD (see Table 5). Unfortunately, we lacked sufficient evidence to make generalizations pertaining to specific VCI subtypes.

**Table 5:** Main electrophysiological signatures for VCI population against healthy control and AD.

**Type of analysis**	**VCI vs. HC**	**VCI vs. AD**
Spectral analysis	• “Slowness” in electrophysiological pattern. Higher power in the δ, θ, and lower in β band associated with the severity of the vascular disease. Lower α power.	• The “slowness” pattern is stronger for VCI with a similar cognitive profile. VCI exhibits an abnormal distribution of sources in slow bands. No definite conclusion for α band, highly sensitive for dementia, but not specific of this pathology.
Connectivity analysis	• Parieto-frontal and interhemispheric disconnection in slow bands (δ, θ, and low α)	• Not robust evidence could be drawn as there is only one paper.
Evoked response	• Slower brain response, accompanied in general with smaller amplitudes, in the responses evoked in visual and auditory tasks.	• No significant differences were found in the literature.
Entropy/ complexity	No definitive conclusion can be drawn for VCI because of the diversity of analysis used.	

### 4.1 Spectral and connectivity VCI electrophysiological patterns

During resting state, the most common pattern observed in patients with VCI is the typical dementia profile known as “slowness” (Rossini et al., 2007, Stam and van Straaten, 2012). This pattern consists of a decline in brain activity that oscillates at higher frequencies and an increase in activity oscillating at lower frequencies. The alpha peak, usually found around 10 Hz, is typically used as the cut point-off between both brain activity regimes. This slowness is present on both spectral and FC studies. The majority of studies showed increased power in slow bands, δ and θ, compared to HC. This increment was directly related to the severity of both cognitive impairment and vascular damage. The pattern appears stronger for patients with VCI than AD with similar cognitive profiles; and VCI patients exhibit greater abnormalities in source distribution within these bands. During functional connectivity analyses, parieto-frontal disconnection, and interhemispheric connectivity in slow bands (δ, θ and low-α) are described for VCI patients versus HC. The most affected area seems to be the parietal area, where there is also the loss of an out-degree hub. Different biological mechanisms have been proposed to explain these slow wave impairments. The correlation between δ power and cerebrovascular severity found in Moretti et al. (2007), even in the absence of cognitive differences, could suggest that a relationship between δ power and cerebrovascular disease exists independently of cognitive impairment. When looking both at intracellular recordings and EEG studies, slow oscillations (especially δ) seem to be mainly generated by the interaction between cortical and subcortical structures (most notably, the thalamus) (Steriade, 2006). In this way, it makes sense that subcortical lesions, commonly the result of vascular damage, would result in this abnormality, affecting both power and connectivity. The increase in δ band power with the severity of cerebrovascular damages could be one of the manifestations of a progressive disconnection caused by the conduction slowing along cortico-subcortical pathways (Moretti et al., 2007). The θ power increment may appear later in the progression of VCI, explaining why it was not apparent in some of the studies.

Decreased power in the β band was also commonly described in patients with VCI compared to HC, and negatively correlated with disease severity. Power in the β band has been suggested to mediate spontaneous cognitive operations during conscious rest (Laufs et al., 2003). The decrease in β power could be related to the impairment of functional cognitive networks, which could explain the differences between groups and the relationship with the degree of cognitive impairment.

Many studies focused on α activity. However, the results were heterogeneous, in great part due to an inconsistent definition of this band and the related sub-bands. Nevertheless, the general trend seems to point towards a decrease in alpha power. When different sub-bands were considered, higher frequencies decreased first when comparing controls to minor VCI, and further for VaD. Lower frequencies seemed to show a less linear pattern. These results could support the more broadly reported slowness of the brain activity towards the theta band. Similar results were found for AD patients. This α power decrement has been previously associated with cholinergic deafferentation (Babiloni et al., 2006a, Babiloni et al., 2006b, Holschneider et al., 1998). This type of deficit is a typical characteristic of AD, where it has been related to degenerative neuronal loss in the basal forebrain (Sarter and Bruno, 2004, Sarter and Bruno, 2002, Sarter and Bruno, 1999, Sarter and Bruno, 1997). However, a cholinergic deficit could also be caused by subcortical cerebrovascular damage to the cholinergic corticospinal pathways (Lim et al., 2020, Wang et al., 2009). Some studies have specifically shown that white matter hyperintensities could be related to the anatomical tracks of the cholinergic pathways (Behl et al., 2007, Shin et al., 2012). Consistent results for AD and VCI patients reinforce the idea that the most widespread findings in VCI are also seen in other dementias, and not necessarily specific to vascular pathology.

### 4.2 Event-Related response for VCI

Using event-related response analyses, VCI patients exhibit slower and reduced brain responses compared to HC, consisting of smaller amplitude responses with longer latencies across visual and auditory tasks. When VCI and AD patients were compared, few differences emerged, although VCI patients showed more significantly diminished amplitudes and longer latencies for P3, N100 and P450 components in comparison to AD patients. Patterns are mostly described in frontal, central and parietal areas. These are also the nodes that appear most impaired when performing connectivity analyses, suggesting a relationship between both characteristics. Furthermore, the connectivity impairments in the studies that analyzed tasks were mainly found in the window near 300 ms, basically the same latency in which the P3 component is found. The latency of the responses was also delayed with respect to HC. Previous studies on cognitive dynamics showed that P3 oscillatory responses are composed mainly of delta and theta bands (Güntekin and Başar, 2010), which were the most affected bands, both in power and in connectivity. It therefore appears that results are consistent across different types of analysis and could represent different “symptoms'' of a single, underlying impaired biological mechanism. As a preliminary hypothesis, such a biological mechanism may be the cortico-thalamo-cortical pathways, which, as previously stated, have been related to these frequencies. Damage to the subcortical regions of the pathway, especially affecting frontal and central nodes, which are more vulnerable than other parts of the brain, may result in the aforementioned changes.

### 4.3 Entropy and complexity measures in VCI

No definitive conclusions can be drawn regarding entropy and complexity in patients with VCI. Most likely, the diversity of methods used has led to seemingly contradictory results. Efforts to replicate the results are needed to determine consistency between samples.

## 5 Conclusion

MEG and EEG quantitative analysis are precise, non-invasive tools with high temporal resolution that reflect changes in bioelectrical activity of the brain. This provides investigators the opportunity to study brain function and network disruption due to changes in synaptic potentials produced by vascular alterations before structural changes and/or cognitive decline are evidenced, as well as the ability to serve as a prognostic tool for disease severity. In addition, it may allow for us the ability to correctly classify VCI and its subtypes. Despite the current limitations, patterns have already emerged, demonstrating the utility of functional analysis to complement and augment structural imaging studies. Further work is needed.

a) The typical dementia profile known as “slowness” is found for VCI patients with increased power in slow bands: delta and theta; and decreased power in the beta band, related to disease severity compared to healthy controls. This pattern seems stronger for VCI than AD with similar cognitive profiles, and VCI patients present a more abnormal source distribution in these bands.
b) A significant parieto-frontal disconnection and reduction in interhemispheric connectivity in slow bands (delta, theta, and low alpha) is described for VCI patients compared to healthy controls. There was not robust evidence found for differences in connectivity between VCI and AD.
c) Longer latencies in brain responses and decreased amplitudes in the evoked responses of VCI patients is seen compared to controls across different tasks (visual and auditory).

## 6 Future research recommendations

In order to establish MEG and EEG as useful biomarkers, a clear definition of VCI and its subtypes is needed. The methodology will need to be standardized, allowing for comparison across groups and consolidation of multicenter efforts. In this context, we propose some future research suggestions, which we hope may lead to electrophysiological signatures being included as complementary information in the future diagnostic criteria of VCI.

1) Operate with homogeneous and consistent criteria for VCI and its subtypes for clinical care and research. Our proposal for further research is to share objective neuroimaging analyses (i.e., to apply automated VCI-related neuroimaging information such as FSL, SPM, or FREESURFER), as well as neuropsychological scores to facilitate VCI research across centers, resulting in larger, homogeneous samples with sociodemographic data, genetic and physiological profiles, and vascular risk factors to allow for better classification of VCI and generalization of its results.
2) Focus research on identification of electrophysiological signatures related to specific cerebrovascular abnormalities (i.e., white matter hyperintensities, lacunar infarcts, brain atrophy, perivascular spaces or cerebral microbleeds. See (Wardlaw et al., 2013). instead of diagnosis groups, as they encompass patients with different cerebrovascular damages. Given the treatable condition of many cerebrovascular diseases, this could lead to treatments aimed at slowing the specific disease process, particularly when considering the mixed dementias. Moreover, this approach will help in early identification of the different subtypes of VCI and improve VaD differentiation from other pure dementias.
3) Include MEG-based studies along with EEG, increasing the ability to detect specific vascular-associated pathology because of the wider sensitive spectral range (Hedrich et al., 2017), and offering a better signal-to-noise ratio (De Jongh et al., 2005) than EEG. However, given EEG is a widely available, low-cost technology, the two should ideally be used as complementary modalities to allow for generalizability and EEG as a first-level scan for brain pathology.
4) Multicentric, multimodal (including electrophysiology), longitudinal and cross-validation studies performed using similar research protocols (e.g., resting, specific task), definitions with respect to frequency band limits and spectrum parameters (e.g., alpha peak and alpha peak amplitude), and methodological signal analysis pipelines (FC or pow approaches) to facilitate reliable, replicable, and reproducible electrophysiological data for the VCI population.

## 7 Data availability

Data sharing is not applicable to this article as no datasets were generated or analyzed during the current study because it is a systematic review of previous literature.

## Funding

This work was supported by the predoctoral researchers grant from Universidad Complutense de Madrid (CT42/18-CT43/18 and CT63/19-CT64/19) and co-founded by Santander bank. Additionally, it was supported by the Ministry of science, innovation, and universities of Spain (FPU18/05768). Finally, Dr. Marsh’s research is supported in part by the American Heart Association and National Institutes of Health

## CRediT authorship contribution statement

**Lucía Torres-Simón:** Conceptualization, Methodology, Formal analysis, Investigation, Writing – original draft, Writing – review & editing, Visualization, Project administration, Funding acquisition. **Sandra Doval:** Conceptualization, Methodology, Formal analysis, Investigation, Writing – original draft, Funding acquisition. **Alberto Nebreda:** Methodology, Formal analysis, Investigation, Writing – original draft, Funding acquisition. **Sophia J. Llinas:** Writing – review & editing, Supervision. **Elisabeth B. Marsh:** Writing – review & editing, Supervision, Validation. **Fernando Maestú:** Writing – review & editing, Resources, Supervision, Funding acquisition.

## Declaration of Competing Interest

The authors declare that they have no known competing financial interests or personal relationships that could have appeared to influence the work reported in this paper.
