# Supplementary material for: Understanding brain function in vascular cognitive impairment and dementia with EEG and MEG: A systematic review
Source: Neuroimage Clin. 2022 May 10;35:103040. doi: 10.1016/j.nicl.2022.103040 (PMC9163840; doi:10.1016/j.nicl.2022.103040)
Supplement: Supplementary data 1 [file mmc1.docx]

**Appendix A. Quality Assessment**

Three reviewers (LT, SD and AN) independently assessed the quality of the articles selected using the Tool for cross-sectional studies using biomarker data (BIOCROSS) (Wirsching et al., 2018). The specific evaluation of the items was adapted, as some items could not be easily applied due to the nature of the research field and electrophysiological biomarkers. These specifications did not modify the scale structure or the aim of study for each item. The changes only attempted to clarify the quality standards in agreement with the study population (i.e., VCI diagnosis criteria) and neurophysiology technical specifications, research protocol or data processing and modeling. Detailed description of each original item **(bold)** and our specifications *(italics)* are reported here below. Final scores for the 32 articles in each quality item and for the three reviewers in the second round are reported in Appendix table 1.

**1st Domain: Study rational**

**Item 1: Hypothesis / Objective**

- 1. **Was the biomarker under study described?**

*Biomarker Description: Explanation in the introduction not only that EEG is used, but how it is related to VCI and what it could mean*.

- 1. **Was the rationale for the study (research question) clearly presented?**

*Why was the study carried out: Description of the research purpose, how the study contributes to previous literature and why it is important and interesting?*

- 1. **Were the study objectives/ hypothesis clearly stated?**

*Objectives/Hypothesis:* *Specific description of objective or hypothesis (It can be to find differences between groups in different variables, without specifying direction). If it is exploratory, it must be specified very clearly.*

**2nd Domain: Design/Methods**

**Item 2: Study Population Selection**

- 1. **Were the characteristics of the study participants presented?**

*Participant Characteristics or basic demographics data of the sample: Age, gender, and specific characteristics of each group, well explained and clear.*

- 1. **Were the disease stages or comorbidities of the included participants described?**

*Disease Stages and Comorbidities: First, clear, and detailed description of the pathology of each group, with severity and comorbidities. Second, the precise type of VCI must be specified or it should be possible to extract it from the diagnostic criteria or data provided (e.g., neuroimaging and neuropsychological scores).*

- 1. **Were the inclusion and exclusion criteria for study participation defined?**

*Inclusion and Exclusion Criteria: Meaningful inclusion or exclusion criteria must be specified. Avoid major biases (e.g., one of the samples being specified as VCI, but then also having a lot of other comorbidities or problems not excluded).*

**Item 3: Study Population Representativeness**

- 1. **Was the sampling frame reported (study population source)?**

*Population Source: Specify where and how the sample was obtained.*

- 1. **Was the participation rate reported (i.e., eligible persons at least 50%)?**

*Participation Rate: Specify the percentage of participation.*

- 1. **Was sample size justification or power description provided?**

*Sample Size justification: Make justification for the sample size. Ideally at the beginning but is also valid at the end if they report why the sample size is sufficient for the research aim.*

**3rd Domain: Data analysis**

**Item 4: Study Population Characteristics**

- 1. **Were the study population characteristics (i.e., demographic, clinical and social) presented?**

*Population characteristics: Specific description of demographic variables and other characteristics of the sample with their corresponding descriptive data.*

- 1. **Were the exposures and potential confounders described?**

*Exposures and Confounders Described: Description of possible confounding variables and description about them in the sample or if they have excluded these subjects.*

- 1. **Were any missing values and strategies to deal with missing data reported?**

*Missing Values: Missing values must be reported and justify.*

**Item 5: Statistical Analysis**

- 1. **Did the authors clearly report statistical methods used to calculate estimates (e.g., Spearman/Pearson/Linear regression, etc.)?**

*Statistical Methods: Report the statistical analysis used in detail. Assess, as far as possible, that these analyses are correctly used (i.e., Assumption of normality, N very small for the method, other assumptions)*

- 1. **Were key potential confounding variables measured and adjusted statistically in reported analyses?**

*Confounding Variables Adjusted: Include possible confounding variables in the analysis or show that there are no differences between possible important confounding variables (Nor non-significant marginal differences, if the N is small).*

- 1. **Was the raw effect size estimate (correlation coefficient, beta coefficient) or measure of study precision provided (e.g., confidence intervals, precise (p-value*)?**

*Effect Size or Study Precision: Explicitly report the values ​​of the statistics and the specific p or effect sizes. If it is a table with many data, allow asterisks with p <0.05 and p <0.01.*

**4th Domain: Data interpretation**

**Item 6: Interpretation & Evaluation of Results**

- 1. **Was the data discussed in the context of study objectives/hypotheses?**

*Data discussed with hypothesis: Results discussed in the context of the objectives and initial hypotheses. Interpretations must be relatively correct and fit the data and hypotheses.*

- 1. **Was the interpretation of the results considering findings from similar studies?**

*Interpretation with other studies: The results should be related with previous studies if possible, and critiques should be reported to explain possible differences if necessary.*

- 1. **Was the biological context described?**

*Biological context described: It should be explained how the results obtained can be interpreted within the underlying biological context (not just saying that there are differences in signals between groups).*

**Item 7: Study Limitations**

- 1. **Was the cross-sectional nature of the analysis discussed?**

*Limitations clear: Some mention to limitations. If they do not mention limitations that we have evidenced in the paper should be evidenced in the quality assessment.*

- 1. **Did the authors acknowledge restricted interpretation due to measurements at one point in time and no statement about causality possible using cross-sectional studies?**

*Restricted interpretation/ No causality: No explicit mention to causality, nor statement that could suggest it. It must be clear that only relationships can be established.*

- 1. **Did the authors acknowledge need for consistency with another research?**

*Replication or further studies: The representativeness of the sample should be discussed. Discuss if the data obtained has already been replicated in other studies, or if more research is needed.*

**5th Domain: Biomarker measurement**

**Item 8: Specimen Characteristics & Assay Methods**

- 1. **Were the measurement methods described? (Assay methods, preservation and storage, detailed protocol, including specific reagents or kits used)**

*Measurement methods: Resting or task design specifications, set-up specifications (i.e., empty room) and data processing.*

- 1. **Were the reproducibility assessments performed for evaluating biomarker stability?**

*Reproducibility assessments: Description in detail of every step followed in the study so that it can be replicated. Specifically, VCI specifications should be well report, to make it truly replicable. If they replicate their measurements to confirm that the signal acquisition has good quality.*

- 1. **Were the quantitation methods well described?**

*Quantification methods: Technique specifications (i.e., number and placement of electrodes, sampling frequency).*

**Item 9: Laboratory Measurement**

- 1. **Was the laboratory/place of measurement mentioned?**

*Laboratory settings: Place where the study was carried out. Setting details and characteristics. Specifically report the laboratory because they can change their characteristics or mode of operation, especially important in multicentric studies.*

- 1. **Were any quality control procedures and results reported (e.g., reported coefficient of variation?**

*Quality control: Explanation of how good their methods are (i.e., in MEG it could be to analyze how good the reconstruction of sources is). Some assessment of quality of the data.*

- 1. **Were the analyses blinded for laboratory staff?**

*Analysis blinded: They must specify something about blinded analysis of the data.*

**Item 10: Biomarker Data Modeling**

- 1. **Was the distribution of biomarker data reported (if non-normal how it was standardized)?**

*Distribution reported: Normality tests should be reported, an image or an explicit description of the distribution of the statistics used. Standardize the data, or use methods that do not require normality, stating it explicitly.*

- 1. **Did the authors report on methods or outlier detection and handling?**

*Outlier detection and handling: Measures must be specified to detect outlier values and say what they do with them (i.e., missed artifacts or subjects, not the filters for the signal).*

- 1. **Were any possible errors resulting from measurement inaccuracies discussed?**

*Measurement inaccuracies: Possible errors arising from the method of signal analysis or MEG/EEG measurement (i.e., signal noise) should be discussed.*

Appendix table 1 Final Quality Assessment for the articles included in the systematic review

| **Study Short Reference** | **Total Score** | **1st Domain: Study rational** | | | | **2nd Domain: Design/Methods** | | | | | | | | **3rd Domain: Data analysis** | | | | | | | | **4th Domain: Data interpretation** | | | | | | | | **5th Domain: Biomarker measurement** | | | | | | | | | | | |
| --- | --- | --- | --- | --- | --- | --- | --- | --- | --- | --- | --- | --- | --- | --- | --- | --- | --- | --- | --- | --- | --- | --- | --- | --- | --- | --- | --- | --- | --- | --- | --- | --- | --- | --- | --- | --- | --- | --- | --- | --- | --- |
|  |  | **Item 1:  Hypothesis / Objective** | | | | **Item 2: Study  Population Selection** | | | | **Item 3:  Study Population Representativeness** | | | | **Item 4: Study Population Characteristics** | | | | **Item 5: Statistical Analysis** | | | | **Item 6: Interpretation & Evaluation of Results** | | | | **Item 7: Study Limitations** | | | | **Item 8: Specimen Characteristics & Assay Methods** | | | | **Item 9: Laboratory Measurement** | | | | **Item 10: Biomarker Data Modeling** | | | |
|  |  | 1 | 1.1 | 1.2 | 1.3 | 2 | 2.1 | 2.2 | 2.3 | 3 | 3.1 | 3.2 | 3.3 | 4 | 4.1 | 4.2 | 4.3 | 5 | 5.1 | 5.2 | 5.3 | 6 | 6.1 | 6.2 | 6.3 | 7 | 7.1 | 7.2 | 7.3 | 8 | 8.1 | 8.2 | 8.3 | 9 | 9.1 | 9.2 | 9.3 | 10 | 10.1 | 10.2 | 10.3 |
| **Al-Qazzaz et al., 2017 (b)** | 9 | 2 | 1 | 1 | 1 | 1 | 1 | 0 | 0 | 1 | 1 | 0 | 0 | 1 | 1 | 0 | 0 | 0 | 0 | 0 | 0 | 0 | 0 | 0 | 0 | 0 | 0 | 0 | 0 | 1 | 1 | 0 | 1 | 1 | 1 | 1 | 0 | 2 | 1 | 1 | 1 |
|  | 10 | 2 | 1 | 1 | 1 | 1 | 1 | 0 | 0 | 1 | 1 | 0 | 0 | 1 | 1 | 0 | 0 | 1 | 1 | 0 | 0 | 0 | 0 | 0 | 0 | 0 | 0 | 0 | 0 | 1 | 1 | 0 | 1 | 1 | 1 | 1 | 0 | 2 | 1 | 1 | 1 |
|  | 10 | 2 | 1 | 1 | 1 | 1 | 1 | 0 | 0 | 1 | 1 | 0 | 0 | 1 | 1 | 0 | 0 | 1 | 1 | 0 | 0 | 0 | 0 | 0 | 0 | 0 | 0 | 0 | 0 | 1 | 1 | 0 | 1 | 1 | 1 | 1 | 0 | 2 | 1 | 1 | 1 |
| **Al-Qazzaz et al., 2017 (a)** | 11 | 1 | 1 | 1 | 0 | 1 | 1 | 0 | 0 | 1 | 1 | 0 | 0 | 1 | 1 | 0 | 0 | 1 | 1 | 0 | 0 | 1 | 0 | 1 | 0 | 2 | 1 | 1 | 1 | 1 | 1 | 0 | 1 | 1 | 1 | 1 | 0 | 1 | 0 | 1 | 1 |
|  | 12 | 1 | 1 | 1 | 0 | 1 | 1 | 0 | 0 | 1 | 1 | 0 | 0 | 1 | 1 | 0 | 0 | 1 | 1 | 0 | 0 | 1 | 0 | 1 | 0 | 2 | 1 | 1 | 1 | 1 | 1 | 0 | 1 | 1 | 1 | 1 | 0 | 2 | 1 | 1 | 1 |
|  | 11 | 1 | 1 | 1 | 0 | 1 | 1 | 0 | 0 | 1 | 1 | 0 | 0 | 1 | 1 | 0 | 0 | 1 | 1 | 0 | 0 | 1 | 0 | 1 | 0 | 2 | 1 | 1 | 1 | 1 | 1 | 0 | 1 | 1 | 1 | 1 | 0 | 1 | 0 | 1 | 1 |
| **Babiloni et al., 2004 (a)** | 14 | 2 | 1 | 1 | 1 | 2 | 1 | 1 | 1 | 0 | 0 | 0 | 0 | 1 | 1 | 1 | 0 | 1 | 1 | 1 | 0 | 2 | 1 | 1 | 1 | 1 | 0 | 1 | 1 | 2 | 1 | 1 | 1 | 1 | 0 | 1 | 0 | 2 | 1 | 1 | 1 |
|  | 14 | 2 | 1 | 1 | 1 | 2 | 1 | 1 | 1 | 0 | 0 | 0 | 0 | 1 | 1 | 1 | 0 | 1 | 1 | 1 | 0 | 2 | 1 | 1 | 1 | 1 | 0 | 1 | 1 | 2 | 1 | 1 | 1 | 1 | 0 | 1 | 0 | 2 | 1 | 1 | 1 |
|  | 14 | 2 | 1 | 1 | 1 | 2 | 1 | 1 | 1 | 0 | 0 | 0 | 0 | 1 | 1 | 1 | 0 | 1 | 1 | 1 | 0 | 2 | 1 | 1 | 1 | 1 | 0 | 0 | 1 | 2 | 1 | 1 | 1 | 1 | 0 | 1 | 0 | 2 | 1 | 1 | 1 |
| **Babiloni et al., 2004 (b)** | 14 | 2 | 1 | 1 | 1 | 2 | 1 | 1 | 1 | 0 | 0 | 0 | 0 | 1 | 1 | 1 | 0 | 1 | 1 | 1 | 0 | 2 | 1 | 1 | 1 | 2 | 1 | 1 | 1 | 2 | 1 | 1 | 1 | 1 | 0 | 1 | 0 | 1 | 0 | 1 | 1 |
|  | 14 | 2 | 1 | 1 | 1 | 2 | 1 | 1 | 1 | 1 | 0 | 0 | 0 | 1 | 1 | 1 | 0 | 1 | 1 | 1 | 0 | 2 | 1 | 1 | 1 | 2 | 1 | 1 | 1 | 2 | 1 | 1 | 1 | 0 | 0 | 0 | 0 | 1 | 0 | 1 | 1 |
|  | 14 | 2 | 1 | 1 | 1 | 2 | 1 | 1 | 1 | 0 | 0 | 0 | 0 | 1 | 1 | 1 | 0 | 1 | 1 | 1 | 0 | 2 | 1 | 1 | 1 | 2 | 1 | 1 | 1 | 2 | 1 | 1 | 1 | 1 | 0 | 1 | 0 | 1 | 0 | 1 | 1 |
| **Beuzeron-Mangina., 2009** | 5 | 1 | 0 | 1 | 1 | 1 | 0 | 1 | 1 | 0 | 0 | 0 | 0 | 0 | 0 | 0 | 0 | 0 | 0 | 0 | 0 | 1 | 1 | 0 | 1 | 0 | 0 | 0 | 0 | 1 | 1 | 1 | 0 | 0 | 0 | 0 | 0 | 1 | 0 | 1 | 0 |
|  | 5 | 1 | 0 | 1 | 1 | 1 | 0 | 1 | 1 | 0 | 0 | 0 | 0 | 0 | 0 | 0 | 0 | 0 | 0 | 0 | 0 | 1 | 1 | 0 | 1 | 0 | 0 | 0 | 0 | 1 | 1 | 0 | 1 | 0 | 0 | 0 | 0 | 1 | 0 | 1 | 0 |
|  | 5 | 1 | 0 | 1 | 1 | 1 | 0 | 1 | 1 | 0 | 0 | 0 | 0 | 0 | 0 | 0 | 0 | 0 | 0 | 0 | 0 | 1 | 1 | 0 | 1 | 0 | 0 | 0 | 0 | 1 | 1 | 1 | 0 | 0 | 0 | 0 | 0 | 1 | 0 | 1 | 0 |
| **Gasser et al., 2008** | 8 | 1 | 0 | 1 | 0 | 1 | 1 | 0 | 0 | 1 | 1 | 0 | 0 | 1 | 1 | 1 | 0 | 2 | 1 | 1 | 1 | 1 | 0 | 1 | 0 | 0 | 0 | 0 | 0 | 0 | 0 | 0 | 0 | 0 | 0 | 0 | 0 | 1 | 1 | 1 | 0 |
|  | 8 | 1 | 0 | 1 | 1 | 1 | 1 | 0 | 0 | 1 | 1 | 0 | 0 | 1 | 1 | 1 | 0 | 2 | 1 | 1 | 1 | 1 | 0 | 1 | 0 | 0 | 0 | 0 | 0 | 0 | 0 | 0 | 0 | 0 | 0 | 0 | 0 | 1 | 1 | 1 | 0 |
|  | 8 | 1 | 1 | 1 | 0 | 1 | 1 | 0 | 0 | 1 | 1 | 0 | 0 | 1 | 1 | 1 | 0 | 2 | 1 | 1 | 1 | 1 | 0 | 1 | 0 | 0 | 0 | 0 | 0 | 0 | 0 | 0 | 0 | 0 | 0 | 0 | 0 | 1 | 1 | 1 | 0 |
| **Gawel., 2007** | 10 | 2 | 1 | 1 | 1 | 1 | 1 | 1 | 0 | 0 | 0 | 0 | 0 | 0 | 0 | 0 | 0 | 1 | 1 | 0 | 0 | 1 | 0 | 1 | 1 | 0 | 0 | 0 | 0 | 2 | 1 | 1 | 1 | 1 | 0 | 1 | 0 | 2 | 1 | 1 | 1 |
|  | 11 | 2 | 1 | 1 | 1 | 1 | 1 | 1 | 0 | 0 | 0 | 0 | 0 | 0 | 0 | 0 | 0 | 1 | 1 | 0 | 0 | 2 | 1 | 1 | 1 | 0 | 0 | 0 | 0 | 2 | 1 | 1 | 1 | 1 | 0 | 1 | 0 | 2 | 1 | 1 | 1 |
|  | 10 | 2 | 1 | 1 | 1 | 1 | 1 | 1 | 0 | 0 | 0 | 0 | 0 | 0 | 0 | 0 | 0 | 1 | 1 | 0 | 0 | 1 | 0 | 1 | 1 | 0 | 0 | 0 | 0 | 2 | 1 | 1 | 1 | 1 | 0 | 1 | 0 | 2 | 1 | 1 | 1 |
| **Gawel., 2009** | 8 | 2 | 1 | 1 | 1 | 1 | 1 | 1 | 0 | 0 | 0 | 0 | 0 | 0 | 0 | 0 | 0 | 1 | 1 | 0 | 0 | 1 | 0 | 1 | 1 | 0 | 0 | 0 | 0 | 1 | 0 | 0 | 1 | 1 | 0 | 0 | 1 | 1 | 1 | 0 | 0 |
|  | 9 | 2 | 1 | 1 | 1 | 1 | 1 | 1 | 0 | 0 | 0 | 0 | 0 | 0 | 0 | 0 | 0 | 1 | 1 | 0 | 0 | 1 | 0 | 1 | 1 | 0 | 0 | 0 | 0 | 2 | 1 | 1 | 1 | 1 | 0 | 0 | 1 | 1 | 0 | 1 | 0 |
|  | 8 | 2 | 1 | 1 | 1 | 1 | 1 | 1 | 0 | 0 | 0 | 0 | 0 | 0 | 0 | 0 | 0 | 1 | 1 | 0 | 0 | 1 | 0 | 1 | 1 | 0 | 0 | 0 | 0 | 1 | 0 | 0 | 1 | 1 | 0 | 0 | 1 | 1 | 1 | 0 | 0 |
| **Jeong et al., 2001** | 11 | 2 | 1 | 1 | 1 | 1 | 1 | 0 | 1 | 1 | 1 | 0 | 0 | 1 | 1 | 1 | 0 | 0 | 0 | 0 | 0 | 1 | 0 | 1 | 1 | 2 | 1 | 1 | 1 | 1 | 1 | 0 | 0 | 1 | 1 | 0 | 0 | 1 | 0 | 1 | 1 |
|  | 12 | 2 | 1 | 1 | 1 | 1 | 0 | 0 | 1 | 1 | 1 | 0 | 0 | 1 | 1 | 1 | 0 | 1 | 1 | 0 | 0 | 2 | 1 | 1 | 1 | 1 | 1 | 0 | 1 | 1 | 0 | 0 | 1 | 1 | 1 | 0 | 0 | 1 | 0 | 1 | 1 |
|  | 10 | 2 | 1 | 1 | 1 | 1 | 1 | 0 | 1 | 1 | 1 | 0 | 0 | 1 | 1 | 1 | 0 | 0 | 0 | 0 | 0 | 2 | 1 | 1 | 1 | 1 | 1 | 0 | 1 | 0 | 0 | 0 | 0 | 1 | 1 | 0 | 0 | 1 | 0 | 1 | 1 |
| **Jiang et al., 2017** | 12 | 2 | 1 | 1 | 1 | 2 | 1 | 1 | 1 | 1 | 1 | 0 | 0 | 1 | 1 | 1 | 0 | 1 | 0 | 1 | 1 | 1 | 0 | 1 | 1 | 2 | 1 | 1 | 1 | 2 | 1 | 1 | 1 | 0 | 0 | 0 | 0 | 0 | 0 | 0 | 0 |
|  | 13 | 2 | 1 | 1 | 1 | 2 | 1 | 1 | 1 | 1 | 1 | 0 | 0 | 1 | 1 | 1 | 0 | 1 | 0 | 1 | 1 | 2 | 1 | 1 | 1 | 1 | 1 | 0 | 1 | 1 | 1 | 0 | 1 | 1 | 1 | 0 | 0 | 1 | 0 | 1 | 0 |
|  | 11 | 2 | 1 | 1 | 1 | 2 | 1 | 1 | 1 | 1 | 1 | 0 | 0 | 1 | 1 | 1 | 0 | 1 | 0 | 1 | 1 | 2 | 1 | 1 | 1 | 1 | 1 | 0 | 1 | 1 | 1 | 0 | 1 | 0 | 0 | 0 | 0 | 0 | 0 | 0 | 0 |
| **Kim et al., 2001** | 12 | 2 | 1 | 1 | 1 | 1 | 1 | 0 | 1 | 1 | 1 | 0 | 0 | 1 | 1 | 1 | 0 | 1 | 0 | 0 | 1 | 1 | 1 | 1 | 0 | 2 | 1 | 1 | 1 | 1 | 1 | 0 | 0 | 1 | 0 | 1 | 0 | 1 | 0 | 1 | 1 |
|  | 11 | 2 | 1 | 1 | 1 | 1 | 1 | 0 | 1 | 1 | 1 | 0 | 0 | 0 | 0 | 0 | 0 | 1 | 0 | 0 | 1 | 1 | 0 | 1 | 1 | 2 | 1 | 1 | 1 | 1 | 0 | 1 | 1 | 1 | 0 | 1 | 0 | 1 | 0 | 1 | 1 |
|  | 12 | 2 | 1 | 1 | 1 | 1 | 1 | 0 | 1 | 1 | 1 | 0 | 0 | 1 | 1 | 0 | 0 | 1 | 0 | 0 | 1 | 1 | 1 | 1 | 0 | 2 | 1 | 1 | 1 | 1 | 1 | 0 | 0 | 1 | 0 | 1 | 0 | 1 | 0 | 1 | 1 |
| **Liedorp et al., 2009** | 12 | 1 | 1 | 1 | 0 | 1 | 1 | 0 | 1 | 1 | 1 | 1 | 0 | 1 | 1 | 0 | 1 | 1 | 0 | 0 | 1 | 1 | 0 | 1 | 0 | 2 | 1 | 1 | 1 | 1 | 1 | 0 | 1 | 2 | 1 | 1 | 1 | 1 | 1 | 0 | 1 |
|  | 10 | 1 | 1 | 1 | 0 | 1 | 1 | 0 | 1 | 1 | 1 | 0 | 0 | 1 | 1 | 0 | 1 | 0 | 0 | 0 | 0 | 1 | 0 | 1 | 0 | 1 | 1 | 0 | 1 | 1 | 1 | 0 | 1 | 2 | 1 | 1 | 1 | 1 | 0 | 1 | 1 |
|  | 11 | 1 | 1 | 1 | 0 | 1 | 1 | 0 | 1 | 1 | 1 | 1 | 0 | 1 | 1 | 0 | 1 | 1 | 0 | 0 | 1 | 1 | 0 | 1 | 0 | 2 | 1 | 1 | 1 | 1 | 1 | 0 | 1 | 1 | 1 | 0 | 1 | 1 | 0 | 0 | 1 |
| **Lin et al., 2015** | 15 | 1 | 1 | 0 | 0 | 2 | 1 | 1 | 1 | 1 | 1 | 0 | 1 | 1 | 1 | 1 | 0 | 2 | 1 | 1 | 1 | 1 | 0 | 1 | 1 | 2 | 1 | 1 | 1 | 2 | 1 | 1 | 1 | 1 | 1 | 1 | 0 | 2 | 1 | 1 | 1 |
|  | 15 | 1 | 1 | 1 | 0 | 2 | 1 | 1 | 1 | 1 | 1 | 0 | 1 | 1 | 1 | 1 | 0 | 2 | 1 | 1 | 1 | 1 | 0 | 1 | 1 | 2 | 1 | 1 | 1 | 2 | 1 | 1 | 1 | 1 | 1 | 1 | 0 | 2 | 1 | 1 | 1 |
|  | 15 | 1 | 1 | 1 | 0 | 2 | 1 | 1 | 1 | 1 | 1 | 0 | 1 | 1 | 1 | 1 | 0 | 2 | 1 | 1 | 1 | 1 | 0 | 1 | 1 | 2 | 1 | 1 | 1 | 2 | 1 | 1 | 1 | 1 | 1 | 1 | 0 | 2 | 1 | 1 | 1 |
| **Lou et al., 2011** | 14 | 2 | 1 | 1 | 1 | 1 | 1 | 0 | 1 | 1 | 1 | 0 | 0 | 1 | 1 | 1 | 0 | 2 | 1 | 1 | 1 | 2 | 1 | 1 | 1 | 1 | 0 | 1 | 0 | 1 | 1 | 0 | 1 | 1 | 1 | 1 | 0 | 2 | 1 | 1 | 1 |
|  | 13 | 2 | 1 | 1 | 1 | 1 | 1 | 0 | 1 | 1 | 1 | 0 | 1 | 1 | 1 | 1 | 0 | 1 | 1 | 1 | 0 | 2 | 1 | 1 | 1 | 1 | 0 | 1 | 1 | 1 | 1 | 0 | 1 | 1 | 1 | 0 | 0 | 2 | 1 | 1 | 1 |
|  | 14 | 2 | 1 | 1 | 1 | 1 | 1 | 0 | 1 | 1 | 1 | 0 | 0 | 1 | 1 | 1 | 0 | 2 | 1 | 1 | 1 | 2 | 1 | 1 | 1 | 1 | 0 | 1 | 0 | 1 | 1 | 0 | 1 | 1 | 1 | 1 | 0 | 2 | 1 | 1 | 1 |
| **Moretti et al., 2004** | 13 | 2 | 1 | 1 | 1 | 2 | 1 | 1 | 1 | 1 | 1 | 0 | 0 | 1 | 1 | 1 | 0 | 1 | 0 | 1 | 1 | 2 | 1 | 1 | 1 | 1 | 0 | 0 | 1 | 1 | 1 | 1 | 0 | 1 | 0 | 1 | 0 | 1 | 0 | 1 | 1 |
|  | 13 | 2 | 1 | 1 | 1 | 2 | 1 | 1 | 1 | 1 | 1 | 0 | 0 | 1 | 1 | 1 | 0 | 1 | 0 | 1 | 1 | 2 | 1 | 1 | 1 | 1 | 0 | 1 | 1 | 1 | 1 | 1 | 0 | 1 | 0 | 1 | 0 | 1 | 0 | 1 | 1 |
|  | 13 | 2 | 1 | 1 | 1 | 2 | 1 | 1 | 1 | 1 | 1 | 0 | 0 | 1 | 1 | 1 | 0 | 1 | 0 | 1 | 1 | 2 | 1 | 1 | 1 | 1 | 0 | 0 | 1 | 1 | 1 | 1 | 0 | 1 | 0 | 1 | 0 | 1 | 0 | 1 | 1 |
| **Moretti et al., 2007** | 14 | 2 | 1 | 1 | 1 | 2 | 1 | 1 | 1 | 1 | 1 | 0 | 1 | 1 | 1 | 1 | 0 | 1 | 0 | 1 | 1 | 2 | 1 | 1 | 1 | 1 | 0 | 1 | 1 | 2 | 1 | 1 | 1 | 1 | 0 | 1 | 0 | 1 | 0 | 1 | 0 |
|  | 14 | 2 | 1 | 1 | 1 | 2 | 1 | 1 | 1 | 1 | 1 | 0 | 1 | 1 | 1 | 1 | 0 | 1 | 0 | 1 | 1 | 2 | 1 | 1 | 1 | 1 | 0 | 1 | 1 | 2 | 1 | 1 | 1 | 1 | 0 | 1 | 0 | 1 | 0 | 1 | 0 |
|  | 14 | 2 | 1 | 1 | 1 | 2 | 1 | 1 | 1 | 1 | 1 | 0 | 1 | 1 | 1 | 1 | 0 | 1 | 0 | 1 | 1 | 2 | 1 | 1 | 1 | 1 | 0 | 1 | 1 | 2 | 1 | 1 | 1 | 1 | 0 | 1 | 0 | 1 | 0 | 1 | 0 |
| **Neto et al., 2015** | 17 | 2 | 1 | 1 | 1 | 1 | 1 | 0 | 1 | 2 | 1 | 1 | 1 | 2 | 1 | 1 | 1 | 2 | 1 | 1 | 1 | 2 | 1 | 1 | 1 | 2 | 1 | 1 | 1 | 1 | 1 | 0 | 1 | 1 | 1 | 1 | 0 | 2 | 1 | 1 | 1 |
|  | 16 | 2 | 1 | 1 | 1 | 1 | 1 | 0 | 1 | 1 | 1 | 0 | 1 | 2 | 1 | 1 | 1 | 2 | 1 | 1 | 1 | 2 | 1 | 1 | 1 | 2 | 1 | 1 | 1 | 1 | 1 | 0 | 1 | 1 | 1 | 1 | 0 | 2 | 1 | 1 | 1 |
|  | 17 | 2 | 1 | 1 | 1 | 1 | 1 | 0 | 1 | 2 | 1 | 1 | 1 | 2 | 1 | 1 | 1 | 2 | 1 | 1 | 1 | 2 | 1 | 1 | 1 | 2 | 1 | 1 | 1 | 1 | 1 | 0 | 1 | 1 | 1 | 1 | 0 | 2 | 1 | 1 | 1 |
| **Rosengarten et al., 2007** | 8 | 1 | 1 | 0 | 0 | 1 | 1 | 0 | 1 | 0 | 0 | 0 | 0 | 1 | 1 | 1 | 0 | 1 | 1 | 0 | 0 | 1 | 0 | 1 | 1 | 0 | 0 | 0 | 0 | 1 | 1 | 0 | 1 | 1 | 0 | 1 | 0 | 1 | 1 | 0 | 0 |
|  | 8 | 1 | 1 | 0 | 0 | 1 | 1 | 0 | 1 | 0 | 0 | 0 | 0 | 1 | 1 | 1 | 0 | 1 | 1 | 0 | 0 | 1 | 0 | 1 | 1 | 0 | 0 | 0 | 0 | 1 | 1 | 0 | 1 | 1 | 0 | 1 | 0 | 1 | 1 | 0 | 0 |
|  | 8 | 1 | 1 | 0 | 0 | 1 | 1 | 0 | 1 | 0 | 0 | 0 | 0 | 1 | 1 | 1 | 0 | 1 | 1 | 0 | 0 | 1 | 0 | 1 | 1 | 0 | 0 | 0 | 0 | 1 | 1 | 0 | 1 | 1 | 0 | 1 | 0 | 1 | 1 | 0 | 0 |
| **Shejorajpanday et al., 2013** | 14 | 2 | 1 | 1 | 1 | 2 | 1 | 1 | 1 | 0 | 0 | 0 | 0 | 1 | 1 | 1 | 0 | 1 | 1 | 0 | 0 | 1 | 0 | 1 | 0 | 2 | 1 | 1 | 1 | 2 | 1 | 1 | 1 | 1 | 0 | 1 | 0 | 2 | 1 | 1 | 1 |
|  | 14 | 2 | 1 | 1 | 1 | 2 | 1 | 1 | 1 | 0 | 0 | 0 | 0 | 1 | 1 | 1 | 0 | 1 | 1 | 0 | 0 | 1 | 0 | 1 | 0 | 2 | 1 | 1 | 1 | 2 | 1 | 1 | 1 | 1 | 0 | 1 | 0 | 2 | 1 | 1 | 1 |
|  | 14 | 2 | 1 | 1 | 1 | 2 | 1 | 1 | 1 | 0 | 0 | 0 | 0 | 1 | 1 | 1 | 0 | 1 | 1 | 0 | 0 | 1 | 0 | 1 | 0 | 2 | 1 | 1 | 1 | 2 | 1 | 1 | 1 | 1 | 0 | 1 | 0 | 2 | 1 | 1 | 1 |
| **Shejorajpanday et al., 2014** | 14 | 2 | 1 | 1 | 1 | 2 | 1 | 1 | 1 | 1 | 0 | 0 | 1 | 1 | 1 | 1 | 0 | 1 | 1 | 0 | 0 | 1 | 0 | 1 | 1 | 1 | 0 | 1 | 1 | 2 | 1 | 1 | 1 | 1 | 0 | 1 | 0 | 2 | 1 | 1 | 1 |
|  | 15 | 2 | 1 | 1 | 1 | 2 | 1 | 1 | 1 | 1 | 0 | 0 | 1 | 1 | 1 | 1 | 0 | 1 | 1 | 0 | 0 | 2 | 1 | 1 | 1 | 1 | 0 | 1 | 1 | 2 | 1 | 1 | 1 | 1 | 0 | 1 | 0 | 2 | 1 | 1 | 1 |
|  | 15 | 2 | 1 | 1 | 1 | 2 | 1 | 1 | 1 | 1 | 0 | 0 | 1 | 1 | 1 | 1 | 0 | 1 | 1 | 0 | 0 | 2 | 1 | 1 | 1 | 1 | 0 | 1 | 1 | 2 | 1 | 1 | 1 | 1 | 0 | 1 | 0 | 2 | 1 | 1 | 1 |
| **Sun et al., 2013** | 11 | 2 | 1 | 1 | 1 | 2 | 1 | 1 | 1 | 1 | 1 | 0 | 0 | 1 | 0 | 1 | 0 | 1 | 0 | 0 | 1 | 1 | 0 | 1 | 1 | 1 | 0 | 0 | 1 | 2 | 1 | 1 | 1 | 0 | 0 | 0 | 0 | 0 | 0 | 0 | 0 |
|  | 12 | 2 | 1 | 1 | 1 | 2 | 1 | 1 | 1 | 1 | 1 | 0 | 0 | 1 | 1 | 1 | 0 | 1 | 0 | 0 | 1 | 2 | 1 | 1 | 1 | 1 | 0 | 0 | 1 | 2 | 1 | 1 | 1 | 0 | 0 | 0 | 0 | 0 | 0 | 0 | 0 |
|  | 11 | 2 | 1 | 1 | 1 | 2 | 1 | 1 | 1 | 1 | 1 | 0 | 0 | 1 | 1 | 1 | 0 | 1 | 0 | 0 | 1 | 1 | 0 | 1 | 1 | 1 | 0 | 0 | 1 | 2 | 1 | 1 | 1 | 0 | 0 | 0 | 0 | 0 | 0 | 0 | 0 |
| **Tsuno et al., 2004** | 11 | 1 | 0 | 1 | 1 | 1 | 1 | 0 | 0 | 1 | 1 | 0 | 0 | 1 | 1 | 1 | 0 | 1 | 0 | 1 | 1 | 2 | 1 | 1 | 1 | 1 | 1 | 0 | 1 | 1 | 1 | 0 | 0 | 1 | 0 | 1 | 0 | 1 | 0 | 1 | 1 |
|  | 11 | 1 | 0 | 1 | 1 | 1 | 0 | 1 | 0 | 1 | 1 | 0 | 0 | 1 | 1 | 1 | 0 | 1 | 0 | 0 | 1 | 2 | 1 | 1 | 1 | 1 | 1 | 0 | 0 | 1 | 1 | 0 | 1 | 1 | 1 | 0 | 0 | 1 | 0 | 0 | 1 |
|  | 12 | 2 | 1 | 1 | 1 | 1 | 1 | 0 | 0 | 1 | 1 | 0 | 0 | 1 | 1 | 1 | 0 | 2 | 1 | 1 | 1 | 1 | 1 | 0 | 0 | 1 | 1 | 0 | 1 | 1 | 1 | 0 | 0 | 0 | 0 | 0 | 0 | 2 | 1 | 1 | 1 |
| **Van Harten et al., 2006** | 11 | 1 | 0 | 1 | 1 | 2 | 1 | 1 | 1 | 0 | 0 | 0 | 0 | 1 | 1 | 1 | 0 | 1 | 0 | 1 | 1 | 1 | 1 | 1 | 0 | 1 | 1 | 0 | 1 | 1 | 1 | 1 | 0 | 2 | 1 | 1 | 1 | 1 | 0 | 1 | 1 |
|  | 12 | 2 | 1 | 1 | 1 | 2 | 1 | 1 | 1 | 0 | 0 | 0 | 0 | 1 | 1 | 1 | 0 | 1 | 0 | 1 | 1 | 2 | 1 | 1 | 1 | 1 | 1 | 0 | 1 | 1 | 1 | 1 | 0 | 1 | 1 | 0 | 1 | 1 | 1 | 1 | 0 |
|  | 11 | 2 | 1 | 1 | 1 | 2 | 1 | 1 | 1 | 0 | 0 | 0 | 0 | 1 | 1 | 1 | 0 | 1 | 0 | 1 | 1 | 1 | 1 | 1 | 0 | 1 | 1 | 0 | 1 | 1 | 1 | 1 | 0 | 1 | 0 | 1 | 1 | 1 | 0 | 1 | 1 |
| **Van Straaten et al., 2012** | 17 | 2 | 1 | 1 | 1 | 2 | 1 | 1 | 1 | 1 | 1 | 0 | 1 | 1 | 1 | 1 | 0 | 2 | 1 | 1 | 1 | 2 | 1 | 1 | 1 | 2 | 1 | 1 | 1 | 2 | 1 | 1 | 1 | 1 | 1 | 0 | 0 | 2 | 1 | 1 | 1 |
|  | 17 | 2 | 1 | 1 | 1 | 2 | 1 | 1 | 1 | 1 | 1 | 0 | 1 | 1 | 1 | 1 | 0 | 2 | 1 | 1 | 1 | 2 | 1 | 1 | 1 | 2 | 1 | 1 | 1 | 2 | 1 | 1 | 1 | 1 | 1 | 0 | 0 | 2 | 1 | 1 | 1 |
|  | 17 | 2 | 1 | 1 | 1 | 2 | 1 | 1 | 1 | 1 | 1 | 0 | 1 | 1 | 1 | 1 | 0 | 2 | 1 | 1 | 1 | 2 | 1 | 1 | 1 | 2 | 1 | 1 | 1 | 2 | 1 | 1 | 1 | 1 | 1 | 0 | 0 | 2 | 1 | 1 | 1 |
| **Van Straaten et al., 2015** | 17 | 1 | 1 | 1 | 0 | 2 | 1 | 1 | 1 | 2 | 1 | 1 | 1 | 2 | 1 | 1 | 1 | 2 | 1 | 1 | 1 | 1 | 0 | 1 | 1 | 2 | 1 | 1 | 1 | 2 | 1 | 1 | 1 | 1 | 1 | 1 | 0 | 2 | 1 | 1 | 1 |
|  | 16 | 1 | 1 | 1 | 0 | 2 | 1 | 1 | 1 | 2 | 1 | 1 | 1 | 2 | 1 | 1 | 1 | 2 | 1 | 1 | 1 | 1 | 0 | 1 | 1 | 1 | 1 | 0 | 1 | 2 | 1 | 1 | 1 | 1 | 1 | 1 | 0 | 2 | 1 | 1 | 1 |
|  | 16 | 1 | 1 | 1 | 0 | 2 | 1 | 1 | 1 | 2 | 1 | 1 | 1 | 2 | 1 | 1 | 1 | 2 | 1 | 1 | 1 | 1 | 0 | 1 | 1 | 1 | 1 | 0 | 1 | 2 | 1 | 1 | 1 | 1 | 1 | 1 | 0 | 2 | 1 | 1 | 1 |
| **Wang et al., 2014** | 8 | 1 | 0 | 1 | 1 | 1 | 1 | 0 | 0 | 1 | 1 | 0 | 0 | 1 | 1 | 0 | 0 | 1 | 1 | 0 | 1 | 1 | 0 | 1 | 1 | 0 | 0 | 0 | 0 | 1 | 1 | 0 | 1 | 0 | 0 | 0 | 0 | 1 | 1 | 1 | 0 |
|  | 8 | 1 | 0 | 1 | 1 | 1 | 1 | 0 | 0 | 1 | 1 | 0 | 0 | 1 | 1 | 1 | 0 | 1 | 1 | 0 | 1 | 1 | 0 | 1 | 1 | 0 | 0 | 0 | 0 | 1 | 1 | 0 | 1 | 0 | 0 | 0 | 0 | 1 | 1 | 1 | 0 |
|  | 8 | 1 | 0 | 1 | 1 | 1 | 1 | 0 | 0 | 1 | 1 | 0 | 0 | 1 | 1 | 1 | 0 | 1 | 1 | 0 | 1 | 1 | 0 | 1 | 1 | 0 | 0 | 0 | 0 | 1 | 1 | 0 | 1 | 0 | 0 | 0 | 0 | 1 | 1 | 1 | 0 |
| **Wang et al., 2016** | 13 | 2 | 1 | 1 | 1 | 1 | 1 | 0 | 1 | 1 | 1 | 0 | 0 | 1 | 1 | 1 | 0 | 1 | 1 | 0 | 0 | 1 | 1 | 1 | 0 | 2 | 1 | 1 | 1 | 1 | 1 | 0 | 1 | 1 | 1 | 1 | 0 | 2 | 1 | 1 | 1 |
|  | 13 | 2 | 1 | 1 | 1 | 1 | 1 | 0 | 1 | 1 | 1 | 0 | 0 | 1 | 1 | 1 | 0 | 1 | 1 | 0 | 0 | 2 | 1 | 1 | 1 | 1 | 1 | 0 | 1 | 1 | 1 | 0 | 1 | 1 | 1 | 1 | 0 | 2 | 1 | 1 | 1 |
|  | 13 | 2 | 1 | 1 | 1 | 1 | 1 | 0 | 1 | 1 | 1 | 0 | 0 | 1 | 1 | 1 | 0 | 1 | 1 | 0 | 0 | 1 | 1 | 1 | 0 | 2 | 1 | 1 | 1 | 1 | 1 | 0 | 1 | 1 | 1 | 1 | 0 | 2 | 1 | 1 | 1 |
| **Wu et al., 2014** | 12 | 2 | 1 | 1 | 1 | 1 | 1 | 0 | 1 | 1 | 1 | 0 | 0 | 1 | 1 | 1 | 0 | 1 | 1 | 1 | 0 | 2 | 1 | 1 | 1 | 1 | 0 | 1 | 0 | 1 | 1 | 0 | 0 | 1 | 1 | 0 | 0 | 1 | 1 | 0 | 0 |
|  | 12 | 2 | 1 | 1 | 1 | 1 | 1 | 0 | 1 | 1 | 1 | 0 | 0 | 1 | 1 | 1 | 0 | 1 | 1 | 1 | 0 | 2 | 1 | 1 | 1 | 1 | 0 | 1 | 1 | 1 | 1 | 0 | 0 | 1 | 1 | 0 | 0 | 1 | 1 | 0 | 0 |
|  | 11 | 2 | 1 | 1 | 1 | 1 | 1 | 0 | 1 | 1 | 1 | 0 | 0 | 1 | 1 | 1 | 0 | 1 | 1 | 1 | 0 | 2 | 1 | 1 | 1 | 0 | 0 | 0 | 0 | 1 | 1 | 0 | 0 | 1 | 1 | 0 | 0 | 1 | 1 | 0 | 0 |
| **Xu et al., 2011** | 9 | 2 | 1 | 1 | 1 | 1 | 0 | 0 | 1 | 1 | 1 | 0 | 0 | 0 | 0 | 0 | 0 | 0 | 0 | 0 | 0 | 1 | 1 | 1 | 0 | 1 | 0 | 0 | 1 | 1 | 1 | 0 | 1 | 1 | 1 | 1 | 0 | 1 | 0 | 1 | 0 |
|  | 9 | 2 | 1 | 1 | 1 | 1 | 0 | 0 | 1 | 1 | 1 | 0 | 0 | 0 | 0 | 0 | 0 | 0 | 0 | 0 | 0 | 1 | 1 | 0 | 1 | 1 | 0 | 0 | 1 | 1 | 1 | 0 | 1 | 1 | 1 | 0 | 0 | 1 | 0 | 1 | 0 |
|  | 9 | 2 | 1 | 1 | 1 | 1 | 0 | 0 | 1 | 1 | 1 | 0 | 0 | 0 | 0 | 0 | 0 | 0 | 0 | 0 | 0 | 1 | 0 | 1 | 0 | 1 | 0 | 0 | 1 | 1 | 1 | 0 | 1 | 1 | 1 | 1 | 0 | 1 | 0 | 1 | 0 |
| **Xu et al., 2012** | 10 | 2 | 1 | 1 | 1 | 1 | 1 | 0 | 1 | 1 | 1 | 0 | 0 | 1 | 1 | 0 | 0 | 0 | 0 | 0 | 0 | 1 | 1 | 1 | 0 | 1 | 0 | 0 | 1 | 1 | 1 | 0 | 1 | 1 | 1 | 1 | 0 | 1 | 0 | 1 | 1 |
|  | 10 | 2 | 1 | 1 | 1 | 1 | 1 | 0 | 1 | 1 | 1 | 0 | 0 | 1 | 1 | 0 | 0 | 0 | 0 | 0 | 0 | 1 | 1 | 1 | 0 | 1 | 0 | 0 | 1 | 1 | 1 | 0 | 1 | 1 | 1 | 1 | 0 | 1 | 0 | 1 | 1 |
|  | 10 | 2 | 1 | 1 | 1 | 1 | 1 | 0 | 1 | 1 | 1 | 0 | 0 | 1 | 1 | 0 | 0 | 0 | 0 | 0 | 0 | 1 | 1 | 1 | 0 | 1 | 0 | 0 | 1 | 1 | 1 | 0 | 1 | 1 | 1 | 1 | 0 | 1 | 0 | 1 | 1 |
| **Xu et al., 2015** | 12 | 2 | 1 | 1 | 1 | 1 | 1 | 0 | 0 | 1 | 1 | 0 | 0 | 1 | 1 | 1 | 0 | 1 | 1 | 0 | 1 | 2 | 1 | 1 | 1 | 0 | 0 | 0 | 0 | 1 | 1 | 0 | 1 | 1 | 1 | 1 | 0 | 2 | 1 | 1 | 1 |
|  | 11 | 2 | 1 | 1 | 1 | 1 | 1 | 0 | 0 | 1 | 1 | 0 | 0 | 1 | 1 | 1 | 0 | 1 | 1 | 0 | 1 | 2 | 1 | 1 | 1 | 0 | 0 | 0 | 0 | 1 | 1 | 0 | 1 | 0 | 1 | 1 | 0 | 2 | 1 | 1 | 1 |
|  | 12 | 2 | 1 | 1 | 1 | 1 | 1 | 0 | 1 | 1 | 1 | 0 | 0 | 1 | 1 | 1 | 0 | 1 | 1 | 0 | 1 | 2 | 1 | 1 | 1 | 0 | 0 | 0 | 0 | 1 | 1 | 0 | 1 | 1 | 1 | 1 | 0 | 2 | 1 | 1 | 1 |
| **Yamaguchi et al., 2000** | 12 | 2 | 1 | 1 | 1 | 1 | 0 | 1 | 1 | 1 | 1 | 0 | 0 | 1 | 1 | 0 | 0 | 1 | 0 | 0 | 1 | 2 | 1 | 1 | 1 | 1 | 0 | 0 | 1 | 1 | 1 | 0 | 0 | 1 | 1 | 1 | 0 | 1 | 0 | 1 | 0 |
|  | 12 | 2 | 1 | 1 | 1 | 1 | 0 | 1 | 1 | 1 | 1 | 0 | 0 | 1 | 1 | 0 | 0 | 1 | 0 | 0 | 1 | 2 | 1 | 1 | 1 | 1 | 0 | 0 | 1 | 1 | 1 | 0 | 0 | 1 | 1 | 0 | 0 | 1 | 0 | 1 | 1 |
|  | 12 | 2 | 1 | 1 | 1 | 1 | 0 | 1 | 1 | 1 | 1 | 0 | 0 | 1 | 1 | 0 | 0 | 1 | 0 | 0 | 1 | 2 | 1 | 1 | 1 | 1 | 0 | 0 | 1 | 1 | 1 | 0 | 0 | 1 | 1 | 0 | 0 | 1 | 0 | 1 | 0 |
